# Supplementary material for: A 6-month, prospective randomized controlled trial of the TargetEd MAnageMent (TEAM) intervention vs. enhanced treatment as usual among Ugandans at risk for stroke
Source: PLoS One. 2025 Aug 22;20(8):e0330606. doi: 10.1371/journal.pone.0330606 (PMC12373184; doi:10.1371/journal.pone.0330606)
Supplement: S2 File — (PDF) [file pone.0330606.s002.pdf]

REDUCING STROKE BURDEN IN UGANDA USING TARGETED MANAGEMENT INTERVENTION (TEAM). A RANDOMISED, PROSPECTIVE 6 – MONTH CONTROLLED TRIAL.

Operational definitions

Stroke - is a medical emergency when the blood supply to part of your brain is interrupted or reduced, preventing brain tissue from getting oxygen and nutrients. Brain cells begin to die in minutes. Early action can reduce brain damage and other complications.

Hypertension – Blood pressure more than 140/90mmHg on two or more occasions

TEAM – Targeted management intervention is a self-management program that helps people to look after themselves and reduce the risk of stroke

Abbreviations

BP – Blood pressure

BMI – Body mass index

ETAU – Treatment as usual

HBA1c – glycosylated hemoglobin

PEDS – peer educator dyads

SAB – stakeholder advisory board

SMP – Self management program

TEAM – Targeted management intervention

Contents

Operational definitions 1
Abbreviations 2
Project Overview: 6
1.0 Introduction 6
1.1 Literature Review 7
1.2 Lack of knowledge regarding stroke is an important risk factor 7
1.3 Practical and effective ways to reduce stroke risk and burden are urgently needed. 8
1.4 Problem statement and Justification. 9
1.5 Study Objectives 9
1.5.1 Overall objective: 9
1.5.2 Specific Objectives 9
2.0 Materials and Methods 10
2.1 Study design and setting 10
2.2 Target population: 10
2.3 Accessible population 10
2.4 Study population: 10
2.5 Participant recruitment 11
2.6 Research participant selection 11
2.6.1 Inclusion criteria 11
2.6.2 Exclusion criteria 11
2.7 Sample size calculation. 11
2.8 Randomization 12
2.9 Study procedures: 12
2.10 Treatment arms. 12
2.10.1 TEAM study arm. 12
2.10.2 CONTROL GROUP - Enhanced Treatment as Usual (ETAU) 13
2.11 Study activities 13
2.11.1 Phase 1 13
2.11.2 Phase 2 14
Quantitative evaluation. 14
Qualitative evaluation. 15
Interventions. 15

Training for TEAM: 16
Peer Dyads: 17
2.12 Schedule of study events. 17
2.13 Measures 17
2.14 Study procedures 18
2.14.1 Blood pressure (BP) 18
2.14.2 Collection of blood for clinical laboratory studies 18
2.14.3 Testing of samples 18
2.15 Primary outcome. 19
2.16 Secondary quantitative outcomes: 19
2.17 Quantitative data Analysis. 19
2.18 Secondary qualitative outcomes: 20
2.19 Missing Data: 21
2.20 Qualitative data analysis plan: 21
2.20.1 Stage 1 21
2.20.2 Stage 2 22
2.21 Data and safety monitoring plan 22
2.21.1 Monitoring entity: 22
Clinicaltrials.Gov Requirements: 23
3.0 DATA MANAGEMENT 24
3.1 Study Data 24
3.2 Data Storage 24
3.3 Quality Control 24
3.4 Data analysis 24
4.0 Ethical considerations 25
4.1 Informed Consent Process: 25
References 29
Appendix A 31
Informed Consent Form - Survey only 31
Appendix B 35
Informed Consent Form - FOCUS GROUPS / INTERVIEWS 35
Appendix C 40
SURVEY - REDUCING STROKE BURDEN IN UGANDA 40

Appendix D – Clinic evaluation 46
Reducing stroke burden in Uganda 46

Project abstract:
Background

Risk factors for stroke (hypertension, obesity, diabetes, tobacco use, sedentary lifestyle, high fat/high salt diet and smoking) are largely modifiable and this presents an opportunity to intervene in reducing stroke burden.

Study objectives:

Objective 1: To refine the TEAM curriculum for optimal acceptability and integration in the Ugandan setting guided by input from stakeholders (patients/family, clinicians, administrators).

Objective 2: To conduct an RCT comparing efficacy of TEAM vs. enhanced treatment as usual (ETAU) in 246 Ugandans (TEAM, N= 123; ETAU, N= 123) at high risk for stroke.

Objective 3: Identify barriers and facilitators to TEAM implementation.

Methods

We propose to conduct a prospective 6-month randomized controlled trial (RCT) in Mulago, Nsambya and Mbarara hospitals that will evaluate the effects of standard medical care + TEAM vs. enhanced medical treatment as usual (ETAU) on key stroke risk factors. Two hundred forty six (246) participants will be randomized at baseline on a 1:1 basis to receive either TEAM (N= 123) or ETAU (N=123). The primary outcome will be BP control, serum cholesterol and blood glucose control. Secondary outcomes of include additional stroke risk biomarkers, (HDL, LDL, triglycerides) diet, exercise, use of alcohol and tobacco, stroke knowledge/attitudes, stress, and treatment adherence with risk-reducing medications. We will assess barriers and facilitators to TEAM implementation using qualitative methods

Data analysis:

We will also explore associations of age, gender, urban vs. rural residential status and stroke history (prior vs. no previous stroke) on TEAM outcomes. Using repeated measures analysis of variance (RMANOVA) we will compare two groups (TEAM intervention vs. enhanced treatment as usual (ETAU)) across three-time waves of systolic BP (H1), serum cholesterol (H2), and serum HbA1c (H3).

Utility:

There is an urgent need to develop an approach that taps into a substantially under-used element of the healthcare system; the power of patients and families to help themselves in reducing stroke burden. This critical to stem the burden and rising numbers of stroke survivors especially in the high-risk populations.

1.0 Introduction

1.1 Literature Review

Stroke is the second commonest cause of mortality worldwide and remains a leading cause of adult disability(1). In Africa, stroke accounts for 15% of hospital admissions and is a major contributor to mortality in both rural and urban areas (2). Stroke is a severely neglected condition in lower-income regions (3, 4). A recent systematic review showed that world-wide stroke incidence declined by 42% in high income countries over the 4 decades from 1970– 1979 to 2000 –2008. During the same period, stroke incidence rose more than 100% in low- to middle-income countries (5). Hypertension is the single most important modifiable stroke risk factor. Worldwide, high blood pressure causes an estimated 7.1 million deaths a year, or approximately 13% of total mortality (6) and according to the World Health Organization, 62% of all strokes are attributable to high blood pressure (4, 7). Compelling clinical trial and epidemiologic evidence suggests that a 10mmhg systolic BP reduction within 3 to 5 years reverses the risk for stroke (8).

In Africa, more than 90% of patients with hemorrhagic stroke and more than half with ischemic stroke have high blood pressure(6). More recent data, such as that from Tanzania, Ghana, Nigeria, Egypt, and South Africa, suggest that hypertension prevalence is on the rise in Africa and commonly exceeds 20%-25% in rural areas and is over 30% in urban and semi-urban areas (6). Our recent study, conducted in Uganda, found a higher prevalence of hypertension (64.9%) among post stroke patients (6, 9) compared to the overall African population with prevalence rates of 5%-20%. We also found a surprisingly high rate of hypertension (54.7%) among non-stroke controls being seen for a variety of other conditions (primarily infection-related)(6, 9, 10). This suggests that hypertension may be more prevalent in Uganda than in other areas of sub-Saharan Africa. In our report, we speculated that this poor blood pressure control might be due to dietary factors. Etiological studies investigating the relationship between sodium intake and stroke have generally produced positive associations. Observational studies show that sustained high daily salt intake (~1 teaspoon) is associated with a 23% greater risk of stroke(11). Excess salt intake is thought to increase stroke risk by increasing blood pressure. A change in sodium intake of 100 mmol/day is associated with a 2.2 mmHg change in systolic blood pressure and 0.1 mmHg for diastolic blood pressure (11, 12). In this same analysis, estimates indicated that reducing sodium intake by 100 mmol/day for ages between 25 and 55 years would reduce stroke mortality by 25%. The International Study of Salt and Blood Pressure (INTERSALT), which included 10,079 subjects from 32 countries, showed a median urinary sodium excretion value of 170 mmol per day (approximately 9.9 g of sodium chloride per day)(13).

1.2 Lack of knowledge regarding stroke is an important risk factor

The lack of correct medical information and poor control of stroke risk factors contributes significantly to the rising incidence of stroke amongst Africans (14, 15). Community attitudes and knowledge influence stroke prevention including risk factor identification and management as well as community and individual response to stroke symptoms when they occur. The success of primary preventive measures and timely medical attention immediately following a stroke is influenced by the public's knowledge and perception of stroke and its risk factors (14, 16). Few studies have been conducted in sub-Saharan Africa, with over half of them in Nigeria. However,

the awareness of stroke, and its risk factors and symptoms are low in community studies conducted in African studies (17, 18). While in Nigerian studies among university staff, students and health workers, the awareness of stroke risk factors was high (14, 15).

In Uganda, the public's understanding and beliefs of stroke, its warning signs and associated risk factors have not been well studied. Nakibuuka and colleagues [10] recently conducted a survey on stroke risk factors and warning signs in community-dwelling individuals. This study [10] found that nearly 3/4 of study participants were unable to identify stroke risk factors and warning signs and did not recognize stroke as a brain disorder (19). Understanding the knowledge gaps and perceptions of stroke are critical to inform and lead to the development of appropriate targeted health promotion campaigns to prevent stroke among high risk populations in our setting. This study assessed the knowledge and perceptions of stroke among urban and rural populations in Mukono district, central Uganda. In addition to replicating elements of the study by Nakibuuka and colleagues, the assessment, as part of larger study on neurological disorder knowledge and attitudes, provided context on how the community perceived neurological disorders more broadly.

1.3 Practical and effective ways to reduce stroke risk and burden are urgently needed.

Risk factors for stroke (hypertension, obesity, diabetes, tobacco use, sedentary lifestyle, high fat/high salt diet and smoking) are largely modifiable and this presents an opportunity to intervene in reducing stroke burden. Reducing stroke risk factors is highly effective in reducing stroke. For example, while every 20/10 mmHg increase in blood pressure (BP) doubles the mortality from stroke, use of antihypertensive therapy can reduce stroke risk by 35-40%.(20, 21). People at high risk for stroke need strategies to reduce risk. For those who have already survived a stroke, stroke recurrence risk increases unless preventative measures are instituted. In Western Uganda, hypertension has been reported in 30.5% of adults over the age of 20 years.(22) Hypertension is a key actionable target for stroke risk reduction in SSA as are other potentially modifiable life-style factors such as obesity and smoking. In spite of the tremendous need, there are few practical and widely implemented care approaches which target stroke burden in SSA. Pilot work by this study team suggests that while many individuals at high risk for stroke know some of their risk factors, they often do not know how to address risk given their own circumstances.(23) Research on interventions to improve BP and other stroke risk outcomes in high-risk individuals in SSA are very limited.(24) Sarfo and colleagues (24) are testing whether an m-Health technology-enabled, nurse-led, multilevel integrated approach is effective in improving blood pressure among Ghanaian stroke patients.

While there are strengths to the approach of Sarfo and colleagues, including use of remote technology and a focus on the individuals at risk for recurrence, the approach does not tap into the ability of patients to become engaged as peers and educators, use family support networks, or substantively address other lifestyle factors for stroke risk. The Stroke Minimization through Additive Anti-atherosclerotic Agents in Routine Treatment (SMAART) trial being conducted in SSA seeks to assess whether a polypill containing 3 antihypertensive agents can impact a stroke vascular biomarker (carotid intimal thickness)(25, 26) However, medications are likely to work best if individuals understand the role of medications and take them in the context of a whole-person approach to risk reduction. An approach that engages people at risk for stroke and which

includes key social and cultural elements may be particularly salient for future generalizability and scale-up.

1.4 Problem statement and Justification.

The stroke burden is severe and growing in SSA, especially Uganda: Stroke is a severely neglected condition in LMICs(3, 4) although it accounts for 15% of hospital admissions and is a major contributor to mortality(2). In SSA, stroke occurs at much early ages, resulting in a greater number of years of potential life lost and worse burden for stroke survivors.(27, 28) Risk factors for stroke which were once rare in traditional African societies, are unfortunately becoming a major public health problem.(29) Western cultural adaptations such as sedentary lifestyle, tobacco and alcohol use, and high fat/salt/cholesterol diets, all increase stroke risk.(28) Population aging and urban migration are also associated with stroke risk factors such as obesity and diabetes. In some SSA countries such as Ghana, South Africa and Cameroon, stroke risk factors have reached epidemic proportions.(30-32) Taken together, stroke prevalence, impact and burden is expected to increase in SSA. There is however, limited information regarding stroke prevention interventions especially among the high-risk populations. Various lifestyle interventions have been developed in the western world, but these are lacking on the African continent where this problem is rising. There are few evidence-based, practical approaches to reduce stroke burden in SSA. There is an urgent need to develop an approach that taps into a substantially under-used element of the healthcare system; the power of patients and families to help themselves in reducing stroke burden. This critical to stem the burden and rising numbers of stroke survivors especially in the high-risk populations. This study will also bridge the literature gap and guide clinical practice in Uganda, by providing tailored information to clinicians and high-risk patients on how to reduce the stroke risk factors.

1.5 Study Objectives

1.5.1 Overall objective:

The overall goal of this 2-phase, 5-year research project is to test an intervention intended to reduce stroke risk factors in Ugandans at high risk for stroke.

1.5.2 Specific Objectives

Objective 1: To refine the TEAM curriculum for optimal acceptability and integration in the Ugandan setting guided by input from stakeholders (patients/family, clinicians, administrators).

Objective 2: To conduct an RCT comparing efficacy of TEAM vs. enhanced treatment as usual (ETAU) in 246 Ugandans (TEAM, N= 123; ETAU, N= 123) at high risk for stroke.

Objective 3: Identify barriers and facilitators to TEAM implementation.

2.0 Materials and Methods

2.1 Study design and setting

This will be a prospective 6-month randomized controlled trial (RCT) in Kiruddu hospital, Mbarara Hospital and Nsambya Hospital.

Kiruddu National Referral Hospital is approximately 13 kilometers (6.1 mi) from Kampala's central business district. Kiruddu National Referral Hospital is the teaching hospital for MakCHS, and has a bed capacity of 400. It provides care to about 80,000 inpatients, and attends to over 250,000 outpatients annually. The hospital has a 40-bed unit which serves in-patients with neurological diseases like stroke. There is also an outpatient clinic for long term follow up of stroke survivors. The neurology inpatient unit admits about 20 patients with acute stroke monthly while the outpatient clinical receives about 120 stroke patients monthly.

Mbarara University of Science and Technology (MUST)/Mbarara hospital. Mbarara Regional Referral Hospital is located in western Uganda, draws clinical volume from the districts of Mbarara, Bushenyi, Ntungamo, Kiruhura, Ibanda, and Isingiro. This location is approximately 265 kilometres (165 mi) southwest of Kampala. Mbarara regional referral Hospital with a bed capacity of approximately 600, serves as the major teaching hospital for MUST. It is a public hospital, founded by the Uganda Ministry of Health, and general care in the hospital is free. The hospital is staffed by medical students and residents. It receives about 150-200 acute strokes/year. Among stroke cases, with 65% ischaemic and 35% hemorrhagic strokes, over 50% have high blood pressure. Mbarara regional referral hospital offers outpatient services to between 1500 - 2000 patients daily, including over 300 at the HIV clinics. There is a dedicated neurology outpatient clinic with majority of these patients being stroke survivors.

St Francis Hospital Nsambya is located 4.8 km south west of Kampala and is a teaching hospital for Uganda Martyrs University. It is a tertiary referral hospital with a capacity of 361 beds. It is involved in patient care, research and teaching. It serves as an "Internship Hospital" for graduates of any of Uganda's four medical schools, where medical graduates spend a year of internship, 3 months in each of the four specialties (Obstetrics and Gynecology, Medicine, Surgery, Pediatrics) under the supervision of specialists and/or consultants. Nsambya Hospital runs general and specialized medical outpatient clinics that receive about 300 outpatients daily of whom 60 -80 attend the neurology clinic where 30-40 patients are stroke survivors. Nsambya Hospital also receives 8–10 new stroke patients per month

2.2 Target population:

All adults at risk for stroke in Uganda

2.3 Accessible population

All adults at risk for stroke presenting to Kiruddu, Mbarara and Nsambya Hospitals.

2.4 Study population:

Adult at risk for stroke, presenting to Kiruddu, Mbarara and Nsambya Hospitals during the study period and meet the inclusion criteria.

2.5 Participant recruitment

Participants will be drawn from the urban medical clinics at Kiruddu National Referral Hospital, the suburban clinics at St. Francis's hospital Nsambya in Kampala, and Mbarara Regional Referral Hospital in Western Uganda. Mbarara Regional Referral Hospital draws from a broad population, including substantial numbers of rural residents.

2.6 Research participant selection

2.6.1 Inclusion criteria

1. Adults ≥ 18 years,
2. At risk for stroke defined by the following;
    a. High systolic BP >140 mmHg (assessed on at least 2 occasions at least 3 days apart and either criterion b or c as noted below:
    b. History of at least 1 other modifiable stroke risk factor including: diabetes, hyperlipidemia, obesity, smoking, alcohol dependent or sedentary lifestyle.
    c. History of stroke or transient ischemic attack within the past 5 years
3. Able to provide written informed consent to participate in the study.

2.6.2 Exclusion criteria

1. Individuals with sickle-cell disease (SCD)
2. Pregnant or lactating women,
3. Individuals with dementia using the Identification for Dementia in Elderly Africans (IDEA).

2.7 Sample size calculation.

From our pilot data, mean baseline systolic BP was 162.9 (SD 25.6). The change from baseline to 6 months with the TEAM Uganda intervention saw a mean systolic BP of 149.7 (SD 22.1). We observed difference in systolic BP from baseline to 24 weeks of 13.22, with standard deviation of 25.84, and within subject correlation parameter value of 0.57. However, we will conservatively estimate our sample size based upon a difference of 10 mmHg, commensurate with a meta-analysis that used this magnitude of change in BP across a large sample with varying baseline BP levels and comorbidities.

Also, in our pilot work with the TEAM Uganda study, we observed an attrition rate of 12.5% at 6-months follow-up. Conservatively, for this RCT, we will assume 25% attrition. Finally, to assume for two-sided test of time-by-treatment group interaction, Type I error level is 0.05. Then, in a linear mixed model with subject-level and center-level random effects, power is 0.80 for a clinically significant difference of 10.0 mm Hg between treatment arms for the projected sample size of 246 subjects in total. We should thus have sufficient power. We do assume compound symmetry over the 3 time periods. We will also consider an AR (1) covariance model, and compare model fits.

Our projected sample size is n=246, with 123 participants per arm.

2.8 Randomization

After obtaining written, informed consent and following all screening and study baseline procedures, individuals will be randomized on a 1:1 basis to participate in either the intervention arm (TEAM) or treatment as usual (ETAU) groups. Block randomization with block sizes ranging randomly between 4 and 8 consecutive patients will be employed to ensure that equal numbers of TEAM and ETAU patients occur within strata and are balanced with respect to relevant comorbidity (diabetes and previous stroke). The randomization list will be computer-generated by personnel within the biostatistics core of the CWRU Neurological and Behavioral Outcomes Center who are not members of the study staff.

2.9 Study procedures:

Individuals who meet the initial screening criteria will be invited to a screening continuation visit in which blood pressure will be assessed again. Those individuals who have a systolic blood pressure ≥ 140 mm Hg on 2 occasions at least 3 days apart will have further assessments, including: serum lipids/glucose levels, HIV serology, review of medical history and medical burden evaluation, review of lifestyle/social factors such as smoking, alcohol and drug use, physical activity, and dietary habits, as well as height and weight evaluation, and current use of medications, such as anti-hypertensives and lipid lowering drugs.

Once eligible participants are confirmed to meet study inclusion criteria, they will complete a baseline evaluation. Baseline evaluation will include demographic variables and existing medical burden (assessed with the self-reported Charlson Index) will be evaluated at baseline, prior to study randomization. Baseline medical status will also be evaluated with personal and family stroke history as well as currently prescribed medications. Primary outcome of the SMP will be change in systolic BP from baseline to 6-month follow-up. Additional outcomes of interest will include diastolic BP, cholesterol/lipids and glycosylated hemoglobin (HbA1c), body mass index (BMI), measures that evaluate diet, activity levels, substance use, self-efficacy, stroke knowledge, stress, medication adherence, medication access and health resource use. Quantitative measures will be repeated at 13-week and 24-week (6-month) and 12 months follow-up except for laboratory testing, which will be conducted only at baseline and 24-week follow-up. Qualitative evaluation will be conducted at baseline and at 13-week follow-up. We will assess each RCT research participant at 12 months follow up (after completion of the TEAM group sessions).

2.10 Treatment arms.

2.10.1 TEAM study arm.

Similar to a previous self-management program (SMP) developed by these investigators this SMP is informed by principles of social cognitive theory. The SMP uses nurses and peer educator dyads (PEDs) composed of patients with stroke risk factors and their care partners to co-deliver an

intervention intended to help reduce future stroke risk. Team begins with one 60-minute 1:1 orientation session, in which the nurse educator and a PED meet with the TEAM participant and his/her care partner.

This is followed by 6 hour-long group sessions with the 6-8 patients and their care partners held approximately weekly. To reinforce learning after the TEAM group sessions are done, 3 brief (approximately 10-20 minute) monthly telephone calls occur between the nurse and the patient over the next 3 months. These calls support ongoing self-management and facilitate linkage with other care providers. All TEAM participants continue in treatment with their regular medical care providers. All TEAM visits will take place in the outpatient clinic where the patient get his/her medical care.

Feasibility and Fidelity: Attendance for each TEAM and ETAU contact session will be recorded. Acceptability will be assessed at 13-weeks follow-up with a brief self-rated survey. Following Fraser et al.,78; fidelity to the TEAM intervention will be assessed quantitatively and qualitatively. Fidelity to TEAM processes, content and format will be evaluated by random attendance of 20% of sessions by non-interventionist study staff to determine if sessions covered relevant TEAM constructs and health practices as identified in the specific sessions as well as assessment of if written prompts were appropriately utilized, and if sufficient time was devoted to the question/answer/comment session. Each fidelity dimension will be rated on a 1-10 scale using the same evaluation process as in the TEAM pilot.

2.10.2 CONTROL GROUP - Enhanced Treatment as Usual (ETAU)

The treatment as usual (ETAU) will consist of an orientation visit (approx. 30 minutes) with a nurse who will provide patient-education materials on stroke risk adapted from the American Heart Association materials and cover common risk factors such as hypertension, obesity, high salt/high fat diet and diabetes. This visit will take place in the outpatient clinic where patients get their medical care. Participants will also receive basic written information in their language of preference and tailored to the reading level of most patients at the clinic. Participants will be offered the opportunity to bring a family member with them to this visit who may also ask questions and who can assist them with understanding written materials for those with limited literacy. To control for the same number of patient contacts as TEAM, the nurse in ETAU will then follow-up with participants with a series of 9 brief phone calls spaced out over the course of 6 months (approximately every 2 weeks during months 1 and 2, then approximately monthly thereafter). Content will reinforce materials provided in the orientation visit and the nurse will be available to answer questions that may arise. Different nursing personnel will deliver TEAM and ETAU interventions to minimize chance of contamination across study arms.

2.11 Study activities

2.11.1 Phase 1

In Phase 1 (months 1-12), we will refine the TEAM intervention for content and process guided by stakeholders (patients/family, clinicians, administrators) in the local context. A stakeholder

advisory board (SAB) will guide modest refinement of the program to meet diverse stakeholder needs. Building upon strong existing partnerships between members of the study team and healthcare partners in Uganda, the study team will obtain input from a stakeholder's advisory board (SAB) to refine the TEAM intervention content to meet the needs of patients and professional healthcare stakeholders and suggest how TEAM might best be incorporated into clinical workflow. The study team has an extensive track -record of convening similar stakeholder advisory groups for self-management interventions in diverse brain disorders. The SAB will be composed of up to 15 relevant stakeholders including 3 stroke survivors, 3 individuals with multiple stroke risk factors as defined above, 3 family members, 3 clinicians and 3 administrators who practice in the proposed study enrollment sites. Consistent with i-PARIHS framework, stakeholders will represent intervention recipients as well as the inner context and outer context of implementation efforts. Patient, family and clinician/administrator representation will be balanced across recruitment sites. There will be 3 video-conference calls during the first 6 months of the project (using Zoom or similar videoconferencing). In the first call, SAB members will review the TEAM curriculum and identify content areas that they feel may need to edited or added. The study team members will then make these modifications to the TEAM intervention manual. It is expected that at least some of the added content will be appropriate for compiling a supplemental content "toolkit" that can be used as needed based on patient needs. In the 2nd meeting, SAB members will review the revised TEAM content and make any additional /final suggestions. In the 3rd meeting, the SAB will be asked to identify strategies that will be helpful to integrating TEAM into clinic workflow. SAB meetings will be audio recorded and assessed qualitatively.

2.11.2 Phase 2

In Phase 2 (months 13-60) we will conduct a prospective, randomized effectiveness-implementation trial of TEAM vs. enhanced medical treatment as usual (ETAU) on key stroke risk factors in Ugandans at high risk for stroke. Two hundred forty-six (N=246) individuals at high risk for stroke will be enrolled, 123 in TEAM and 123 in ETAU. The project will be conducted across 3 sites in Uganda (Kiruddu National Referral Hospital and outpatient clinics, and health centers associated with Nsambya Hospital and Mbarara Regional Referral Hospital) that will enroll a representative sample of Ugandans at risk for stroke. The primary intent of the study is to assess whether TEAM is better able to reduce stroke risk factors compared to ETAU among Ugandans at high risk for stroke. If findings are positive, the study team will then plan a strategy for broader scale-up of the intervention. A unique feature of TEAM is the use of an individual who has had a stroke and his or her care partner (the peer dyad) to help teach and support individuals who are at high risk for stroke in better managing their health. The total number of participants for this project will be 246 patient participants in the RCT, up to 12 peer educators, and up to 12 family members/care partners of peer educators (total of 270 individuals)

Quantitative evaluation.

Since stroke is a moderately long-term health outcome (years to decades) that typically occurs in the presence of one or more stroke risk factors, the project will focus on testing whether TEAM Uganda can modify well-established short-term biomarkers that predict stroke risk, specifically blood pressure control, serum total cholesterol and blood glucose control. Secondary outcomes of interest include additional stroke risk biomarkers (HDL, LDL, and triglycerides), diet, exercise,

use of alcohol and tobacco, stroke knowledge, stress, medication adherence, and participant satisfaction with TEAM and with ETAU. The RCT will evaluate key primary (systolic BP) and secondary (biological and behavioral) outcomes indicative of stroke risk. H1: Individuals in TEAM will have significantly reduced systolic BP compared to individuals randomized to ETAU. H2: Individuals randomized to TEAM will have greater reductions in serum cholesterol compared to ETAU. H3: Individuals with diabetes randomized to TEAM will have improved glycemic control as measured by serum HbA1c compared to ETAU. Secondary outcomes also include other biomarker variables, stroke knowledge and attitudes, medication adherence and health resource use. We will also explore associations of age, gender, urban vs. rural residential status and stroke history (prior vs. no previous stroke) on TEAM outcomes.

Alcohol dependency will be assessed using the AUDIT screening tools with questions such as; frequency, type of alcohol and quantity consumed. Participants will be classified as alcohol dependent if they exceed the recommended level for safe alcohol intake i.e. more than 3 drinks on average every time they drink, or if they undertook binge drinking (i.e. more than 3 drinks on one occasion in the one month preceding the evaluation (33, 34).

Qualitative evaluation.

To better understand the barriers and facilitators to stroke risk reduction that need to be further integrated into TEAM and evaluate how the RCT sample might compare to the sample enrolled in previous pilot work, the study team will conduct qualitative interviews for individuals who are at risk for stroke. Interviews will use a semi-structured guide adapted from the instrument used in the TEAM study. Qualitative informants will include 30 participants from the TEAM group and 30 patients from the ETAU group (total N=60). The sample will be distributed in approximately equal proportions across the 3 study sites. We will balance the selection of respondents by age, gender and diabetes comorbidity. The interviews will be audio-recorded, transcribed verbatim, and then translated into English. Final transcripts will have all personal identifiers or specific information that could identify any specific individual removed. In addition to the baseline qualitative evaluation, a representative sample of RCT participants will be evaluated (N= 30 in TEAM, N=15 in ETAU) at the 13-week time point to assess their perceptions of stroke risk factor management, what elements of TEAM or ETAU they might have found particularly helpful, and what could be improved in future efforts targeting stroke risk factor reduction. We will also conduct qualitative interviews with 5 clinic staff at each site (N=15) to derive information on process measures relevant to future TEAM implementation (barriers, facilitators, complementarity with clinic workflow.

Interventions.

Nurses and peer educator dyads (PEDs) composed of individuals who have had success in managing stroke risk factors and one individual in their support system that the person identifies as key to their health management) will be trained to deliver TEAM. In the previous TEAM pilot, all care partners were family members. For this RCT, the study team will hire and train up to 8 nurse educators and up to 12 PEDs. The TEAM program uses nurses and peer educator dyads (PEDs) composed of patients and their care partners to co-deliver an intervention intended to help

reduce future stroke risk (Figure 1). Team begins with one 60-minute 1:1 orientation session, in which the nurse and PED meet with the patient and his/her care partner. This is followed by a one-hour-long group sessions with 6-8 patients and their care partners held approximately weekly. Our pilot work suggests that the timing and format of the groups is highly acceptable to participants, with robust attendance/retention. Individuals who miss a group session are encourage to complete a make-up session conducted immediately before or after regularly scheduled groups. In cases where logistic barriers prevent in-person attendance, participants may conduct make-up sessions by phone with the nurse educator. To reinforce learning after the TEAM group sessions are done, 3 brief (approximately 10-20 minute) monthly telephone calls occur between the nurse and the patient over the next 3 months. These calls support ongoing self-management and facilitate linkage with other care providers. All TEAM participants continue in treatment with their regular medical care providers and TEAM visits will take place in these clinics.

Training for TEAM:
The team will hire and train up to 8 nurses and up to 12 PEDs. Training will follow the same procedures as in the TEAM Uganda pilot and in past self-management trials. Initial training will be a 2-day intensive to review the TEAM curriculum followed by training /support sessions monthly during year 1, at least quarterly thereafter, attended by clinical research staff, nurses and PEDs.

Figure 1: Team topics and content

Session 1: Orientation and introductions, Emphasize ground rules, Establish a therapeutic relationship, Discuss facts and myths about stroke, Overview and interactive discussion of stroke risk factors

Session 2: Medications to manage complications and reduce future risk, Nutrition for best physical and emotional health, Healthy cooking and use of salt in food preparation

Session 3: Problem-solving skills and the IDEA approach (Identify the problem, Define possible solutions, Evaluate the solutions, Act on the best solution), Effects of exercise, smoking and other substances in recovery

Session 4: Medication routines, Coping with stress and depression, Making healthy changes

Session 5: The importance of having a regular healthcare provider, Talking with health care providers, Working with traditional healers

Session 6: A personal care plan to take care of the body and mind, Acknowledgement of group progress, Self-management and recovery as a lifestyle

The ETAU group will consist of a single visit (approx. 30-45 minutes) with a nurse who will provide patient- education regarding stroke risk. Content will be adapted from the American Heart Association materials and cover common risk factors such as hypertension, obesity, high salt/high fat diet and diabetes. The nurse will be available for questions on these materials during this visit, which will take place in the outpatient clinic where the patient gets his/her medical care. In addition to the nurse visit, patients will receive basic written information in their language of preference

and tailored to the reading level of most patients at the clinic. ETAU participants will be offered the opportunity to bring a family member with them to this visit to help ask questions and assist them with understanding written materials for those with limited literacy. To control for the same number of patient contacts as TEAM, the nurse in ETAU will then follow-up with patients with a series of 9 brief phone calls spaced out over the course of 6 months (approximately every 2 weeks during months 1 and 2, then approximately monthly thereafter). Content will reinforce materials provided in the orientation visit and the nurse will be available to answer questions that may arise. ETAU participants will be asked to come in for follow-up 13-week, 6- month and 12 months visits

Peer Dyads:
The investigators will enroll up to 12 PEDs. The PED will consist of a patient and his or her care partner. Patient peer educators will be recruited from clinical practices of the co-investigators or from the community and trained using procedures similar to those conducted by these investigators in previous work using peer educators in multiply morbid samples.

To be eligible to participate as peer educator, the individuals must have or have/had the same stroke risk factors as noted in the patient inclusion criteria (hypertension + one other risk factor or past stroke/TIA). As with the peer educators, the care partner/family member will also provide informed consent to study participation. Individuals with known sickle-cell disease, pregnant or lactating women, and individuals with dementia will be excluded from being peer educators.

2.12 Schedule of study events.

Each research participant will be assessed 4 times: at screening, at baseline, at 13 weeks (after completion of the TEAM group sessions), at 6 months' follow-up and at 12 months follow up. During the 12-month (Twelve months) post baseline, we will assess enduring effects of the intervention via a short 5-question survey attached. Qualitative interviews in a sub-set of RCT patient participants (20 at each site, 10 for TEAM and 10 ETAU, total of 60 participants) will be conducted at base-line and 6 months. Qualitative interviews will also be conducted with clinic staff (N=5 at each site, total of 15 participants) and will represent differing types of personnel staffing the clinics. Participants will be reimbursed $15 for each quantitative screening, baseline, and 13-week interview and $20 for the final (6-month) interview. All assessments for a single participant will be done by a single rater trained by the proposed study PIs to pre-established and documented reliability standards. It is expected that patient assessment will require approximately 60-90 minutes for the screening assessment and approximately 45-60 minutes for the baseline and three follow-up assessments. Participants will be reimbursed $20 for each qualitative interview.

2.13 Measures

Demographic variables and existing medical burden (assessed with the self-reported Charlson Index) will be evaluated at baseline, prior to study randomization. Baseline medical status will also be evaluated with personal and family stroke history as well as currently prescribed medications. Primary outcome of the RCT will be change in systolic BP from baseline to 6-month follow-up. Additional outcomes of interest will include diastolic BP, cholesterol/lipids and glycosylated hemoglobin (HbA1c), body mass index (BMI), measures that evaluate diet, activity levels, substance use, self-efficacy, stroke knowledge, stress, medication adherence, medication access and health resource use. Quantitative measures will be repeated at 13-week and 24 weeks

(6-month) follow-up except for laboratory testing, which will be conducted only at baseline and 24-week follow-up. During the 12-month (Twelve months) post baseline, we will assess enduring effects of the intervention via a short 5-question survey. Qualitative evaluation will be conducted at baseline and at 13-week follow-up.

2.14 Study procedures

2.14.1 Blood pressure (BP)

The study participants will be requested beforehand to refrain from smoking and drinking alcohol or caffeinated beverage half an hour before the examination. Before the blood pressure measurement begins the subjects will be requested to abstain from eating, drinking other than water, smoking and taking drugs that affect blood pressure one hour before measurement. The subjects will be requested to roll up the sleeves of the shirts or blouses so that the upper right arm is bare. Blood pressure will be measured with an Omron automated sphygmomanometer model HEM-907 whose accuracy has been validated (Gurpreet et al). The blood pressure on the left arm will be measured after the participant has sat for at least five minutes, legs uncrossed, the arm resting on the table and the ante cubital fossa at the level of the sternum. Two arm cuffs that fit arm circumferences 9 – 13 inches and 13 -17 inches will be used in the process. Three readings will be taken three minutes apart and the closest two will be used to describe the blood pressure of the patient. Hypertension will be defined as a systolic blood pressure ≥140 mmHg and/or a diastolic blood pressure of ≥90 mmHg or treatment with anti-hypertensive medication in accordance with the United States Seventh Joint National Committee on Detection, Evaluation and Treatment of Hypertension (JNC-VII).

2.14.2 Collection of blood for clinical laboratory studies

On the day of sample collection, a venipuncture will be performed under aseptic conditions and in line with standard procedures to obtain 5mls of blood from each participant at least 8 hours after they have had the last meal and the following tests will be performed:

I. Fasting lipid profile (total cholesterol, triglycerides, HDL and LDL cholesterol)
II. Hemoglobin A1c
III. Fasting blood sugar
IV. HIV testing
V. Treponema Pallidum Antibody Test (TP-PA)
VI. Rapid Plasma Reagin
VII. Serum Creatinine

Blood for fasting blood sugar will be dispensed into a Sodium fluoride (grey top) tube and inverted 12 times. Blood for fasting lipid profiles (Triglycerides, HDL and LDL-cholesterol) will be dispensed into a yellow SST (yellow top) with gel separator. Blood for HIV serology, Treponema pallidum Antibody (TP-PA), Rapid plasma regain and serum creatinine estimation, will be collected into the SSt (red top) and left to clot to obtain serum.

2.14.3 Testing of samples

All the tests will be done at the site using the following techniques: -

Lipid panels on serum from Yellow SST (yellow top): All the lipid profile (triglycerides, HDL- and LDL-cholesterol) and total cholesterol will be done using Retrofron ^{R} dry chemistry kits from Roche Diagnostics.

HIV serology on serum from red top tube: Tests to detect HIV antibodies will be done using HIV rapid strips (Determine Statpak and Unigold) as outlined in the national guidelines for HIV testing from the Ministry of Health. Pre and Posttest counseling for HIV will be done and participants with positive results will be referred for further management at established centers.

Other tests such as TP-PA, RPR, serum creatinine and fasting blood sugar will be performed based on the laboratory SOPS and quality control and assurance protocols.

Loss to follow up

To prevent loss to follow-up we will train the study staff, implement data quality procedures, and we will have scheduled telephone calls to the study participants/ caregivers to remind them of their scheduled visits. Additionally, we included a 15% loss to follow up during the sample size estimation. Additionally, there are statistical methods available to deal with missing data, but these procedures

2.15 Primary outcome.

The primary study outcome is systolic BP measured at baseline, 13-week, 24 weeks (6 month). All BP measurements will adhere to a standardized procedure previously used by this study team.

2.16 Secondary quantitative outcomes:

Secondary biomarker outcomes will include diastolic BP, serum cholesterol and lipids, HbA1c and BMI. Life style changes of interest will include, diet and salt intake as measured by the modified dietary questionnaire(35), physical activity as measured by the Global Physical Activity Questionnaire (GPAQ)(36) , tobacco and alcohol use measured by the Global Adult Tobacco Survey (GATS) questionnaire and The Alcohol Use Disorders Identification Test (AUDIT) respectively(37, 38). To assess risk factor management self-efficacy we will use the General Self-Efficacy measure (35) and stroke knowledge using the same set of questions used in the stroke survey conducted by Nakibuuka and colleagues(39). As a key focus of TEAM is to encourage engagement and adherence with medical management, we will collect information on medications for stroke risk factors (type and total number). Recognizing that adherence can be intentional or non-intentional we will collect data on adherence via use of a standardized adherence attitudinal measure (Medication Adherence Report Scale /MARS)(40, 41) and query individuals on difficulty (financial or otherwise) in taking medications or medical care.

2.17 Quantitative data Analysis.

Preliminary descriptive analyses will examine change over time in systolic BP as well as secondary outcomes. Primary and secondary outcome measurements will be assessed at baseline prior to

randomization to either TEAM or ETAU, and at the 13 week and 6-month time-points using the same methods as in our pilot work. Of primary interest is the treatment by time interaction in systolic BP, measured from baseline to 6 months after randomization between TEAM and ETAU. Explanatory variables will include age, gender, stroke history and rural vs. urban status.

The 13-week systolic BP measurement will reflect the period following baseline (when individuals participating in TEAM Uganda will have just completed the “intensive” group sessions), and it will be used to assess for within-subject differences from baseline to 13 weeks and 13 weeks to 6 -month time periods. This will allow for a greater understanding of the time course for when the expected reductions occur in the TEAM Uganda group, which we expect to mostly occur after the “intensive’ group sessions have ended. We will analyze change from baseline to 13 weeks as well, analyzing contrasts within the mixed model framework.

For aim 2, we will use 2 group by 3 time waves repeated measures analysis of variance (RMANOVA) for each of the 3 hypotheses in Aims 2. These analyses will compare two groups (TEAM intervention vs. enhanced treatment as usual (ETAU)) across three-time waves of systolic BP (H1), serum cholesterol (H2), and serum HbA1c (H3). When using a repeated measures ANOVA, it not only assesses mean differences across time, but also assesses group differences, as well as, the interaction of time X group which will allow us to test the trend of the means over time across the three groups. The repeated measures ANOVA, not only can be used to determine if there are mean differences across the three time periods, it can also utilize orthogonal polynomial contrasts to determine linear and quadratic trends of the means across time. Orthogonal polynomials are weights assigned to each time period that model a linear or quadratic (non -linear) trend. Repeated measures ANOVA can determine if these trends in the means are significant. A linear trend is indicated if there is a steady increase or decline in scores from the first time wave to the third time wave. A quadratic trend is indicated if there is a change in direction based on scores across the time waves. For example, if there is a decrease in mean scores from T1 (baseline) to T2 (13 weeks), but an increase in scores at the T3 (6 months), this would be indicative of a U trend for the three time periods and a return toward the mean score from T1 to T3. A quadratic trend can also indicate a single change in direction in the trend of the means, for example, if there is a decline in means scores from T1 to T2, and the decreased mean score is maintained at T3. The major assumption to be tested with repeated measures ANOVA is sphericity. Sphericity is a form of compound symmetry and refers to the equality of variances of the differences between each time wave. If the assumption of sphericity is violated based on the Mauchly’s test, SPSS provides an adjusted F, df, and p-value in repeated measures ANOVA to account for this violation. We may also explore repeated measures mixed model approaches to analyze change from baseline to 13 weeks as well, analyzing contrasts within the mixed mod el framework.

2.18 Secondary qualitative outcomes:

We will model secondary outcomes over time in a similar manner. We will consider generalized linear models when distributions of outcomes are not approximately normally distributed, or transformations of outcome variables to normality. We will also conduct exploratory moderator analyses of the explanatory variables in the primary mixed model. Mediation analyses also will be

explored. Potential mediators include dose of TEAM session exposure, medication prescription / access and adherence to a heart healthy diet. Variables with (change) values that appear to be associated with change in systolic BP levels and that appear to differ by treatment will be considered further as mediator variables, following as in MacKinnon (2008) and Preacher and Hayes (2008). This will involve single mediator analyses as well as multiple mediator models. These analyses will involve the treatment variable and change in systolic BP values. Associated standard errors of estimated indirect effects will be derived through bootstrapping, using the M-Plus software.

2.19 Missing Data:

Strategies to minimize loss to follow-up are outlined in Form E, specifically the description of our Recruitment and Retention Plan. Data that remain missing despite our retention efforts will be accommodated in our analyses and their impact evaluated through sensitivity analyses. The models we propose can be estimated without bias under the missing at random (MAR) assumption and provide valid analysis as long as covariates associated with missingness (If any) are included in the mixed model. To assess which covariates may be associated with missing outcome data, we will create binary indicators of whether the outcome was missing (=1) or not (=0). If a covariate is correlated with missingness at r >0.40 and is correlated at r >0.40 with the original response variable, it will be included in the analysis as an auxiliary correlate. We will conduct assessment of the missing at random (MAR) assumption by pattern mixture models that relax the missing at random assumption, while analyzing the sensitivity of treatment by time interaction effects. We will also consider using Full Information Maximum Likelihood models in presence of incomplete data.

2.20 Qualitative data analysis plan:

2.20.1 Stage 1.

Two qualitative researchers will be used to ensure standardization of qualitative analysis. The qualitative team will first independently review each transcript and highlight significant statements, sentences, or quotes. Based on review of the independently derived statements, the team will develop consensus-based “clusters of meaning” or relevant “themes and categories”. Each researcher will further read/code each document independently and iteratively until no new insights emerge. Initial codes will be recorded using NVivo. These entries will be elaborated as coding progresses. The qualitative researchers will then construct a consensus-based coding dictionary that includes mutually exclusive definitions for each code. This coding structure will be reviewed after a preliminary analysis of a sub sample of transcripts, and the dictionary will be refined through comparison, categorization, and discussion. Although code definitions are mutually exclusive, the same portions of text can be attached to multiple codes.

The refined codes will then be applied to the transcripts, with coding decisions recorded electronically using NVivo. All transcripts will be coded by both qualitative researchers. In our previous research, checks for inter -rater consistency using Cohen’s Kappa yielded Kappa ≥ .90 which was considered excellent agreement.

2.20.2 Stage 2.

NVivo will be used to retrieve all segments of text attached to a particular code to create code - based files across all respondents. The qualitative team will further elaborate, refine, and differentiate the codes and identify similarities and differences through comparison of respondents. Emergent observations will be recorded in theoretical memos using NVivo's Project Document feature. This process of engagement with the data and iterative discussions will be repeated until all discrepancies are resolved and no new insights emerge.

2.21 Data and safety monitoring plan

2.21.1 Monitoring entity:

The study PIs, Drs. Katabira and Sajatovic, will monitor the study to ensure data integrity and safety of the participants. The MakCHS site Data Coordinator, will review the data for discrepancies on a regular basis and will review the study records for compliance with IRB requirements and verification of source documents. Ugandan investigators and research staff will be responsible for all stages of the project: recruiting and consenting participants, conducting the surveys, qualitative interviews, and conducting all study assessments. They will be responsible for all data collection. Management of data will take place at MakCHS, but de-identified data will be transferred to CWRU for final analysis.

The data safety and monitoring plan for this project consists of several components, as outlined below. The PIs (Dr. Martha Sajatovic & Dr. Elly Katabira) will be responsible for ensuring that this plan is followed during the course of this study.

Approval from the University Hospitals Cleveland Medical Center (UHCMC) Institutional Review Board (IRB) and from the Makerere College of Health Sciences (MakCHS) IRB will be obtained prior to performing any research related to this study and approval will be maintained throughout the study period via continuing review.

Drs. Katabira & Sajatovic will conduct regular research staff meetings to closely monitor study start -up and progress, including oversight of staff training and research capacity building. As was done in the Neurology MEPI and in the R21 pilot study, meetings that involve both Ugandan and U.S. study team members will be held via web teleconference. Meeting minutes will be sent to research staff participants. More frequent local meetings and ad-hoc full team meetings that may be called in the event that problems or concerns arise during the study period will supplement the regularly scheduled web calls.

There will be at least 2 in-person visits to the sites annually by the U.S. PI.

Adverse event identification/classification: The study investigators and/or qualified research assistants will identify adverse events. All adverse events, whether considered serious or not, will be recorded and reviewed by the study PIs on an ongoing basis, and reported to the IRB according to local IRB policy. Serious adverse events are defined as events that result in any of the following: death; a life-threatening experience; inpatient hospitalization or prolongation of existing hospitalization; a persistent or significant disability/incapacity; or a congenital anomaly/birth defect (or an event that may require medical or surgical intervention to prevent one of the outcomes listed above). Strokes will be recorded if/when they occur and will be considered serious adverse

events. All adverse events, including all serious adverse events, will be reported to the IRB according to local IRB policy and to the Data Safety and Monitoring Board (DSMB). A summary report of all adverse events will be submitted to NINDS annually, and at the end of the study.

Data and Safety Monitoring Board (DSMB) and safety review plan: We recognize the need for careful, expert external data and safety oversight to ensure the wellbeing of the participants in this study and the scientific integrity of the project. These experts, who are not members of the study team, will review and evaluate the accumulated data for participant safety, adverse events, study conduct and progress, at minimum, every 12 months. Ad-hoc meetings might be called to evaluate unanticipated serious adverse events or any other urgent issues that are relevant and which might occur during the course of the study. The DSMB will be comprised of two clinicians with stroke expertise at the Uganda site; a faculty member/clinician with stroke expertise at the US site, and a biostatistician at the US site who are all not part of the study team, but have extensive experience with federally funded research. The DSMB communication and oversight will be accomplished via telephone, SKYPE, or email communication for issues that need more immediate attention.

The PIs will review study progress and safety regularly as described above. As noted above, progress reports, including patient recruitment, retention/attrition, and adverse events will be provided to the DSMB at least annually for independent review. There are no specific study stopping rules as the intervention arm will receive self-management information that will help teach and support individuals who are at high risk for stroke in better managing their health. The annual report will include a list and summarization of adverse events. In addition, the annual report will address (1) whether adverse event rates are consistent with pre- study assumptions; (2) reason for dropouts from the study; (3) whether all participants met entry criteria; (4) whether continuation of the study is justified on the basis that additional data are needed to accomplish the stated aims of the study; and (5) conditions whereby the study might be terminated prematurely. There will be an interim analysis conducted once half of all trial participants have completed the 6 -month outcome evaluation point. The annual report will be signed by the PI/designate and will be forwarded to the DSMB. The DSMB will review the annual plan on all 5 points outlined above, as well as the interim analysis, and make recommendations to the appropriate regulatory agencies (IRB, NIH) concerning continuation, modification or termination of the study. The PI will provide a copy of all DSMB reports to the appropriate IRB and NIH on an annual basis.

Clinicaltrials.Gov Requirements:

While this proposal does not involve a drug, biologic therapy, or device, Drs. Katabira and Sajatovic will register the clinical trial on the ClinicalTrials.gov website and Dr. Sajatovic's site will keep the information up to date.

3.0 DATA MANAGEMENT

The PIs, Dr Sajatovic and Dr Katabira, will monitor the study to ensure data integrity and the safety of the participants. The PIs will work closely with the rest of the Ugandan study team to quickly identify any adverse events/safety risks that might occur, manage the events as are clinically indicated, and report promptly to the appropriate regulatory authorizes/IRBs.

The PIs will hold regular weekly meeting with the study staff to review study progress and any issues that may come up regarding adverse events. All adverse events will be reviewed by Dr. Sajatovic, Dr. Katabira and the other members of the Ugandan study team on an ongoing basis. In addition, all adverse events, including all serious adverse events, will be reported to the IRB according to local IRB policy.

3.1 Study Data

Study questionnaires will be used for data collection. Study teams will move in pairs; an interviewer who will administer the questionnaire and an assistant who will record answers on the paper questionnaire. Data captured will be entered into a Microsoft Access database by the data entry clerk and will be double entered to ensure accuracy. Back-up files of the database will be kept at the end of each data entry session. For purposes of quality control, query programs will be written into the database to limit the entry of incorrect data and ensure entry of data into the required fields.

3.2 Data Storage

Source data in this study will include survey questionnaires and results from blood tests. All study documents will be kept in secure filing cabinets in the offices. The principal investigator will be responsible for the security of all study documents.

3.3 Quality Control

All members of the study team will be trained in the project objectives, methods of effective communication with study participants and collection of high-quality data. Study team will receive additional training specific to the tasks that they will perform within the project including the interview techniques, administration of surveys, completing questionnaires. Standard Operating procedures (SOPs) will be written for project activities and booklets of all relevant documents will be provided to each member of the project team.

3.4 Data analysis

All data will be analyzed using STATA version 11 (College Station Texas) and EpiData Analysis (The EpiData Association, Odense, Denmark). Logistic regression analysis will be used to identify socio-behavioral characteristics associated with the most prevalent stroke risk factors in addition to hypertension. Factors significantly associated with stroke risk factors at bi-variable analysis ($P<0.05$) will be included in the adjusted model. Descriptive statistics of mean, frequencies and percentages will be used to summarize and present data on the participants' stroke-related

knowledge, attitudes and perceptions. Bivariate analysis will be used to test associations between demographic/clinical characteristics and attitudes/perceptions. Hypothesis testing will be 2-tailed with a type 1 error set at 0.05. Factors significant at bivariate analysis will undergo multivariate analysis using multivariable logistic regression model. We shall tabulate, score the information and KIIs and FGDs.

4.0 Ethical considerations

4.1 Informed Consent Process:

Approval from local leaders will be sought before the study activities commence in the proposed areas. All study participants will be asked to give their written consent prior to participating in the study activities. A copy of the consent form will be given to the study participant.

VOLUNTARY PARTICIPATION

Participants will be told that enrolling into the study is voluntary and that they are free to withdraw from the study at any time without penalty. The investigator may withdraw a participant without his/her consent if it is in the participant's best interest.

RISKS

There is no physical risk to the participants. This study does not affect clinical care. The risks to the participants will be primarily those of talking about some matters that they may find uncomfortable. There is a risk of loss of confidentiality. As part of this protocol, participants will be tested for HIV (human immunodeficiency virus). They will be notified of the results of the testing, and counseled as to the meaning of the results, whether they are positive or negative. If the test indicates that they are infected with HIV, they will receive additional counseling and referred to HIV care centers for further care and investigation. The test results will be kept confidential to the extent permissible under the law.

The insertion of the needle to draw blood can be painful and blood draws may cause bleeding, bruising, discomfort, infections, dizziness, or fainting.

BENEFITS

There is no guarantee of benefits to any participants. However, subjects may benefit from knowing their risk of stroke and how to reduce the risk of various stroke risk factors using self-management.

CONFIDENTIALITY

Confidentiality of information collected is of fundamental importance. The research team will be trained to adhere to strict confidentiality guidelines.

Complete confidentiality cannot be assured due to the nature of group interactions in the qualitative segment of Phase 1. Participants will be reminded that they should maintain other participants' confidentiality. Confidentiality of research data will be protected in several ways. Patient identifiers at the analytic level will not be the same as the patient's clinic medical record number.

The files that link the patient identifiers to the study numbers will be kept in locked cabinets in the study staff offices. Only aggregate data will be presented or published, and will be presented such that individual patients cannot be identified. The proposed project's research personnel who will have access to participant identities are the study PIs, co-investigators, and the study staff. The research staff will receive training regarding special considerations in interviewing about potentially sensitive topics. As much as possible, assessments will be done in private space.

FINANCIAL CONSIDERATIONS

All participants will receive a stipend to compensate for time and travel. Individuals will receive 50,000/= for the screening assessments, which include the informed consent process for their transport and time compensation. For those who are randomized for long-term follow up they will receive 50,000/= for each subsequent visit and a meal during the training sessions. Participants in the focus groups and individual qualitative interviews will receive 50,000/= and a meal during these sessions. All study subjects will provide informed consent whereby all the benefits and risks that will arise out of their participation in the study are clearly delineated.

Institutional Review Boards

This protocol and related information sheets will be reviewed and approved by all IRBs before the study begins. Any amendments or modifications to the protocol will also be reviewed prior to implementation. The IRBs will include: 1) The School of Medicine Research and Ethics Committee (SOMREC) of the College of Health Sciences, Makerere University; 2) Uganda National Council for Science and Technology (UNCST).

Study monitoring

On-site monitoring will be conducted to ensure that the study is conducted in compliance with applicable regulations and guidelines; is recorded and reported in accordance with the protocol; and is consistent with national guidelines and locally accepted practices and SOPs. The monitor will confirm the quality and accuracy of data at the site by verification against the source documents such as clinical records, consent forms and against the database where applicable. The investigators and volunteers, consenting to participate in the study agree that the monitor may inspect study facilities and source records (e.g. informed consent documents, clinic and laboratory records, other source documents) as well as observe the performance of study procedures. Such information will be treated as strictly confidential and will under no circumstances be made available to the public. The principal investigator will permit inspection of the facilities and all study-related documentation by authorized representatives of government and regulatory authorities relevant to this study.

Table 1: Schedule of study events

PROCEDURES	Screen	Baseline	Wk 1-12	Wk 13	Wk 13 - Mth 6	Mth 6	Mth 12
**Identification of participants:**<br>Inclusion/Exclusion criteria review	X						
Informed Consent	X						
Demographics		X					
Medical Status & Burden:<br>Self-reported Charlson Comorbidity Index<br>Medications<br>Personal and family stroke history		X					
**Randomization**		X					
**Primary Outcome:** Systolic BP		X		X		X	
**Secondary Outcomes** such as (Diastolic BP, Serum cholesterol, HbA1c, Serum HDL, LDL, triglycerides, BMI, Diet questionnaire, GPAQ, GATS, AUDIT, General self-efficacy, Stroke knowledge, INTERSTROKE stress, Medication adherence (MARS), Medication Access and Health resource use)		X		X		X	
**Qualitative Assessment**		X		X			

Barriers & Facilitators to stroke risk management<br><br>Clinic workflow compatibility							
TEAM groups attendance			X				
Follow-up phone calls<br><br>* ETA contact by phone			X*		X		
Acceptability survey				X			
Reach/utility among clinicians				X		X	
**Enduring effects of the intervention**							X

Table 2: Timeline

Activity	Date
Submit study protocol and questionnaires to research ethics committee	August 2020
Approval of final protocol from all research ethics committees including UNCST	September 2020
Produce and finalize SOPs (study and laboratory)	September – October 2020
Train field staff (based on SOPs)	November 2020
Selection of advisory board	November 2020
Finalizing TEAM training materials Phase 1	December 2020 – June 2021
Phase 2 intervention study	July 2021 – July 2022

References

1. Armario P, de la Sierra A. Antihypertensive treatment and stroke prevention: are angiotensin receptor blockers superior to other antihypertensive agents? Ther Adv Cardiovasc Dis. 2009;3(3):197-204.
2. IMAM I, M. Stroke: a review with an African perspective. Annals of TropicalMedicine & Parasitology. 2002;96(5):435-45.
3. O'Donnell M, Yusuf S. Tackling the global burden of stroke: The need for large-scale international studies. Lancet Neurol 2009;8:306-7.
4. Xiu-Yang Li X-LC, Ping-Da Bian & Liu-Ru Hu. High Salt Intake and Stroke: Meta-analysis of the Epidemiologic Evidence. CNS Neuroscience & Therapeutics 2012;18: 691-701.
5. Chin JH. **STROKE IN SUB-SAHARAN AFRICA: AN URGENT CALL FOR PREVENTION**. Neurology. 2012;78:1007-8.
6. Mensah GA. Epidemiology of stroke and high blood pressure in Africa. Heart. 2008;94(6):697-705.
7. Brundtland GH. From the World Health Organization.Reducing risks to health,promoting healthy life. JAMA. 2012;288:1974.
8. Greenlund KJ, Giles WH, Keenan NL, Croft JB, Mensah GA. Physician advice, patient actions, and health-related quality of life in secondary prevention of stroke through diet and exercise. Stroke. 2002;33(2):565-70.
9. Kaddumukasa M, Ddumba E, Duncan P, Goldstein LB. Poststroke hypertension in Africa. Stroke. 2012;43(12):3402-4.
10. Mensah GA. Epidemiology of Stroke and High Blood Pressure in Africa. Heart. 2008;94:697-705.
11. Ding EL, Mozaffarian D. Optimal dietary habits for the prevention of stroke. Semin Neurol. 2006;26(1):11-23.
12. Stamler J, Rose G, Stamler R, Elliott P, Dyer A, Marmot M. INTERSALT study findings. Public health and medical care implications. Hypertension. 1989;14(5):570-7.
13. Adrogue HJ, Madias NE. Sodium and potassium in the pathogenesis of hypertension. N Engl J Med. 2007;356(19):1966-78.
14. Akinyemi RO, Ogah OS, Ogundipe RF, Oyesola OA, Oyadoke AA, Ogunlana MO, et al. Knowledge and perception of stroke amongst hospital workers in an African community. Eur J Neurol. 2009;16(9):998-1003.
15. Obembe AO, Olaogun MO, Bamikole AA, Komolafe MA, Odetunde MO. Awareness of risk factors and warning signs of stroke in a Nigeria university. J Stroke Cerebrovasc Dis.23(4):749-58.
16. Jones SP, Jenkinson AJ, Leathley MJ, Watkins CL. Stroke knowledge and awareness: an integrative review of the evidence. Age Ageing.39(1):11-22.
17. Cossi MJ, Preux PM, Chabriat H, Gobron C, Houinato D. Knowledge of stroke among an urban population in Cotonou (Benin). Neuroepidemiology.38(3):172-8.
18. Wahab KW, Okokhere PO, Ugheoke AJ, Oziegbe O, Asalu AF, Salami TA. Awareness of warning signs among suburban Nigerians at high risk for stroke is poor: a cross-sectional study. BMC Neurol. 2008;8:18.
19. Nakibuuka J, Sajatovic M, Katabira E, Ddumba E, Byakika-Tusiime J, Furlan AJ. Knowledge and Perception of Stroke: A Population-Based Survey in Uganda. ISRN Stroke.2014.

20. Lewington S, Clarke R, Qizilbash N, Peto R, Collins R. Age-specific relevance of usual blood pressure to vascular mortality: a meta-analysis of individual data for one million adults in 61 prospective studies. Lancet. 2002;360(9349):1903-13.
21. Neal B, MacMahon S, Chapman N. Effects of ACE inhibitors, calcium antagonists, and other blood-pressure-lowering drugs: results of prospectively designed overviews of randomised trials. Blood Pressure Lowering Treatment Trialists' Collaboration. Lancet. 2000;356(9246):1955-64.
22. Wamala JF, Karyabakabo Z, Ndungutse D, Guwatudde D. Prevalence factors associated with Hypertension in Rukungiri District, Uganda - A Community-Based Study. African health sciences. 2009;9(3):153-60.
23. Kaddumukasa M, Kayima J, Kaddumukasa MN, Ddumba E, Mugenyi L, Pundik S, et al. Knowledge, attitudes and perceptions of stroke: a cross-sectional survey in rural and urban Uganda. BMC Res Notes. 2015;8:819.
24. Sarfo FS, Treiber F, Jenkins C, Patel S, Gebregziabher M, Singh A, et al. Phone-based Intervention under Nurse Guidance after Stroke (PINGS): study protocol for a randomized controlled trial. Trials. 2016;17(1):436.
25. Sarfo FS, Sarfo-Kantanka O, Adamu S, Obese V, Voeks J, Tagge R, et al. Stroke Minimization through Additive Anti-atherosclerotic Agents in Routine Treatment (SMAART): study protocol for a randomized controlled trial. Trials. 2018;19(1):181.
26. Sarfo FS, Ovbiagele B. Stroke minimization through additive anti-atherosclerotic agents in routine treatment (SMAART): A pilot trial concept for improving stroke outcomes in sub-Saharan Africa. J Neurol Sci. 2017;377:167-73.
27. Lemogoum D, Degaute JP, Bovet P. Stroke prevention, treatment, and rehabilitation in sub-saharan Africa. American journal of preventive medicine. 2005;29(5 Suppl 1):95-101.
28. Feigin VL, Forouzanfar MH, Krishnamurthi R, Mensah GA, Connor M, Bennett DA, et al. Global and regional burden of stroke during 1990-2010: findings from the Global Burden of Disease Study 2010. Lancet. 2014;383(9913):245-54.
29. Agyemang C. Rural and urban differences in blood pressure and hypertension in Ghana, West Africa. Public health. 2006;120(6):525-33.
30. Thorogood M, Connor M, Tollman S, Lewando Hundt G, Fowkes G, Marsh J. A cross-sectional study of vascular risk factors in a rural South African population: data from the Southern African Stroke Prevention Initiative (SASPI). BMC public health. 2007;7:326.
31. Bosu WK. Epidemic of hypertension in Ghana: a systematic review. BMC public health. 2010;10:418.
32. Fezeu L, Minkoulou E, Balkau B, Kengne AP, Awah P, Unwin N, et al. Association between socioeconomic status and adiposity in urban Cameroon. International journal of epidemiology. 2006;35(1):105-11.
33. Organization WH. International guide for monitoring alcohol consumption and related harm 2011 [Available from: http://apps.who.int/iris/bitstream/10665/66529/1/WHO_MSD_MSB_00.4.pdf.
34. Organization WH. WHO STEPS manual, STEPS instrument 2011 [Available from: http://www.who.int/chp/steps/STEPS_Manual.pdf?ua=1.
35. Charlton KE, Steyn K, Levitt NS, Jonathan D, Zulu JV, Nel JH. Development and validation of a short questionnaire to assess sodium intake. Public Health Nutr. 2008;11(1):83-94.
36. World Health Organization. Global Physical Activity Surveillance.

37. Office on Smoking and Health NCfCDPaHP. The Global Tobacco Surveillance System (GTSS).
38. Babor T F H-BJ, Saunders J, Monteiro M, . The Alcohol Use Disorders Identification Test. Guidelines for Use in Primary Care. . Geneva, Switerzland World Health Organisation 2001.
39. Nakibuuka J, Sajatovic M, Katabira E, Ddumba E, Byakika-Tusiime J, Furlan AJ. Knowledge and Perception of Stroke: A Population-Based Survey in Uganda. ISRN Stroke. 2014;2014.
40. Thompson K, Kulkarni J, Sergejew AA. Reliability and validity of a new Medication Adherence Rating Scale (MARS) for the psychoses. Schizophr Res. 2000;42(3):241-7.
41. Nguyen TM, La Caze A, Cottrell N. What are validated self-report adherence scales really measuring?: a systematic review. Br J Clin Pharmacol. 2014;77(3):427-45.

Appendix A

Informed Consent Form - Survey only
INTRODUCTION

You are being requested to take part in this study about identifying risk factors for stroke. The doctor in charge of this study at this site is Dr. Elly Katabira. Before you decide if you want to be a part of this study, we want you to know about the study.

This is a consent form. It gives you information about this study. The study staff will talk with you about this information. You are free to ask questions about this study at any time. If you agree to take part in this study, you will be requested to sign this consent form **first**. You will get a copy to keep.

This study is conducted by investigators from MakCHS, Nsambya, Mbarara University and Case Western Reserve University/UHCMC, Cleveland, OH (USA), and has been approved by both MakCHS and the UHCMC Institutional Review Boards.

WHY IS THIS STUDY BEING DONE?

This study is being done so that we can see what people in Uganda know about stroke and what the risk factors are.

WHAT DO I HAVE TO DO IF I AM IN THIS STUDY?

Once you agree to participate, you will be requested to complete a questionnaire about what you know about stroke. The research staff will measure your blood pressure and they will request you to go to a designated clinic within your area for an additional visit the next morning if your blood pressure is equal or above 140/90.

At the clinic visit, they will measure your blood pressure, and if your blood pressure is equal or higher to 140/90 at the two readings, the study staff will also take a sample of your blood through a vein in your arm (about 5ml or 1tsp). This blood will be used to test for the amount of fats and sugar in the blood, as well as for HIV. The study staff will also ask more questions about

your general health, your medical history, any medications you may be taking and your lifestyle (diet, exercise, alcohol and tobacco use, etc.).

The reason we are asking about your lifestyle, measuring your blood pressure and checking the amount of fats and sugar in the blood, as well as HIV, is because they can all affect the risk of stroke. You will be told the results of the tests and the study staff may refer you to a health clinic for additional information and care, if you have risk factors for stroke.

If your blood pressure is lower than 140/90 when you are at the clinic, the study staff will not collect any more information, and your participation in the study will be over.

All information that is collected for this study will be kept confidential and will only be available to study staff.

HOW MANY PEOPLE WILL BE IN THIS STUDY?

We expect that up to about 246 study participants will be enrolled with 123 in the study arm and 123 in the treatment as usual arm.

HOW WILL YOU GET THE SAMPLES FROM ME?

If you agree to have samples collected, you will have some blood collected from a vein in your arm. The insertion of the needle to draw blood is painful; however, this discomfort is brief. For most people, needle punctures to get blood samples do not cause any serious problems; however, they may cause bleeding, bruising, discomfort, infections, dizziness, or fainting.

As part of this protocol, you will be tested for HIV (human immunodeficiency virus, which is the virus that causes the acquired immunodeficiency syndrome [AIDS]). You will be notified of the results of the testing, and counseled as to the meaning of the results, whether they are positive or negative. If the test indicates that you are infected with HIV, you will receive additional counseling about the significance for your medical care and possible risks to other people. The test results will be kept confidential to the extent permissible under the law. However, you may opt out for the HIV testing.

HOW WILL YOU USE MY SAMPLES?

The samples will be tested and then discarded. The results from the tests will be recorded. The samples will be analyzed at Mulago hospital for your blood fats.

COULD THIS STUDY BE STOPPED EARLY?

This study could be stopped at any time by the IRB or EC at your site, the local or national ministry of health in your country, the ACTG, Office for Human Research Protections (OHRP), the U.S. National Institute of Neurological Disorders and Stroke, or other government agencies, as part of their duties to protect research study participants.

ARE THERE BENEFITS TO TAKING PART IN THIS STUDY?

There are no direct benefits to you. You may benefit from knowing whether or not you may be at risk for stroke. You might also benefit by knowing your blood sugar and fats.

The benefit of doing this research is to learn more about what people know about stroke. This may help other people in the future.

WHAT ARE THE RISKS OF THIS STUDY?

The greatest risk is to your privacy. However, your results will be recorded and kept in a confidential manner and will only be communicated to you or your health providers.

Taking blood may cause some discomfort, bleeding, or bruising where the needle enters the body, lightheadedness, and in rare cases, fainting or infection.

WHAT OTHER CHOICES DO I HAVE BESIDES THIS STUDY?

You may choose not to participate in this study and this will not affect your care.

WHAT ABOUT CONFIDENTIALITY?

We will do everything we can to keep your personal information confidential. Researchers will label your samples with a code. This means that your name or any other information that could directly identify you is replaced with a "code." A list linking your code to your name is kept by the original researchers. This coding makes it harder for other researchers to find out who you are.

We cannot guarantee absolute confidentiality. Your personal information may be disclosed if required by law. No publication of this study will use your name or identify you personally.

People who may review your records include: SOMREC or other local regulatory agencies, National Institutes of Health, Office for Human Research Protections, study staff, study monitors, and their designees.

WHAT ARE MY RIGHTS?

Your participation is entirely voluntary and you are free to take part or withdraw at any time. This will not affect any medical care you may receive now or in the future. You may choose to answer some or all questions posed.

WILL I RECEIVE COMPENSATION?

You will receive UShs. 50,000 for completing the first part of the study, that is the short questionnaire and have your blood pressure measured the first time. You will receive an additional UShs. 50,000 if you come to the clinic for the study sessions. This is to help cover the cost of transportation and to compensate you for time spent on the study.

WHOM DO I CONTACT FOR QUESTIONS?

The interviewer has discussed this information with me and offered to answer my questions. If you have questions about taking part in this research program, or about the

information in this consent form, please contact the Principal Investigator Prof E. Katabira 0712-853410 or Dr. Mark Kaddumukasa 0772633475. You may also contact the Chairman School of Medicine Research and Ethics Committee. Prof Ponsiano Ocama; Tel: 0772421190 if these questions are not answered.

Summary of your rights as a participant in a research study

Your participation in this research study is voluntary. Refusing to participate will not alter your usual health care or involve any penalty or loss of benefits to which you are otherwise entitled. If you decide to join the study, you may withdraw at any time and for any reason without penalty or loss of benefits. If information generated from this study is published or presented, your identity will not be revealed. In the event new information becomes available that may affect the risks or benefits associated with this study or your willingness to participate in it, you will be notified so that you can decide whether or not to continue participating. If you experience physical injury or illness as a result of participating in this research study, medical care is available at the nearest health center or elsewhere; however, the study research has no plans to provide free care or compensation for lost wages or physical injuries or illness.

Disclosure of your study records

Efforts will be made to keep the personal information in your research record private and confidential, but absolute confidentiality cannot be guaranteed. The School of Medicine, Research and Ethics Committee, UNCST and University Hospitals Case Medical Center Institutional Review Board may review your study records. If this study is regulated by the Food and Drug Administration (FDA), there is a possibility that the FDA might inspect your records. In addition, for treatment studies, the study sponsor and possibly foreign regulatory agencies may also review your records. If your records are reviewed your identity could become known.

Contact information

____ has described to you what is going to be done, the risks, hazards, and benefits involved. The Principal Investigator [Prof Elly Katabira] can also be contacted at [0712-853410]. If you have any questions, concerns or complaints about the study in the future, you may also contact them later.

If the researchers cannot be reached, or if you would like to talk to someone other than the researcher(s) about; concerns regarding the study; research participant's rights; research- related injury; or other human subject issues, please call the Chairman School of Medicine Research and Ethics Committee. Prof Ponsiano Ocama; Tel: 0772421190

Signature

Signing below indicates that you have been informed about the research study in which you voluntarily agree to participate; that you have asked any questions about the study that you may have; and that the information given to you has permitted you to make a fully informed and free decision about your participation in the study. By signing this consent form, you do not waive any

legal rights, and the investigator(s) or sponsor(s) are not relieved of any liability they may have. A copy of this consent form will be provided to you.

x	
Signature of Participant	Date
x	
Printed Name of Participant	

x	
Signature of Participant	Date
x	
Printed Name of Participant	

Appendix B

Informed Consent Form - FOCUS GROUPS / INTERVIEWS
INTRODUCTION

You are being requested to take part in this study about identifying risk factors for stroke. The doctor in charge of this study at this site is Dr. Elly Katabira. Before you decide if you want to be a part of this study, we want you to know about the study.

This is a consent form. It gives you information about this study. The study staff will talk with you about this information. You are free to ask questions about this study at any time. If you agree to take part in this study, you will be asked to sign this consent form **first**. You will get a copy to keep.

This study is conducted by investigators at MakCHS and Case Western Reserve University/UHCMC, Cleveland, OH (USA), and has been approved by both MakCHS and the UHCMC Institutional Review Boards.

WHY IS THIS STUDY BEING DONE?

This study is being done so that we can learn what people in Uganda know about stroke and how well they are able to take care of their health.

WHAT DO I HAVE TO DO IF I AM IN THIS STUDY?

Once you agree to participate, you will be requested to participate in a focus group or in a one-on-one interview with study staff.

If you live in a city, and you have hypertension and at least 1 other stroke risk factor, you will be asked to participate in one focus group. The focus groups will be conducted by investigators from MakCHS, and there will be up to 8 individuals per group. During the focus group you will be able to discuss what you feel makes it easy or difficult for you to take good care of your health. The focus groups will be audio recorded, transcribed word for word, and then translated into English. All information that could identify you will be removed.

If you have had a stroke and you were hospitalized for a stroke, study staff will ask you to participate in one-on-one interviews. You will be asked about what you feel makes it easy or difficult for you to take care of yourself and helps you prevent another stroke. The interviews will be audio recorded, transcribed word for word, and then translated into English. All information that could identify you will be removed.

All information that is collected for this study will be kept confidential and will only be available to study staff.

HOW MANY PEOPLE WILL BE IN THIS STUDY

We will enroll 16 participants who have at least two risk factors for stroke for the focus groups. We will enroll 15 participants who have had a stroke for the one-on-one interview.

COULD THIS STUDY BE STOPPED EARLY?

This study could be stopped at any time by the IRB at your site, UNCST, the local or national ministry of health in your country, the ACTG, Office for Human Research Protections (OHRP), the U.S. National Institute of Neurological Disorders and Stroke, or other government agencies, as part of their duties to protect research study participants.

ARE THERE BENEFITS TO TAKING PART IN THIS STUDY?

There are no direct benefits to you. You may benefit from learning more about stroke and about how to take care of your health.

The benefit of doing this research is to learn more about what people know about stroke. This may help other people in the future.

WHAT ARE THE RISKS OF THIS STUDY?

The greatest risk is to your privacy.

WHAT OTHER CHOICES DO I HAVE BESIDES THIS STUDY?

You may choose not to participate in this study.

WHAT ABOUT CONFIDENTIALITY?

We will do everything we can to keep your personal information confidential. Your name or any other information that could directly identify you is replaced with a "code." A list linking your code to your name is kept by the original researchers. This coding makes it harder for other researchers to find out who you are.

We cannot guarantee absolute confidentiality. If you participate in the focus groups, your identity will be known by the other participants. Everybody will be asked to respect confidentiality and to not share information from the other participants with people outside of the study.

Your personal information may be disclosed if required by law. No publication of this study will use your name or identify you personally.

People who may review your records include: (Makerere University *School of Medicine Research and Ethics committee, Case Western Reserve University* Institutional Review Board, other local regulatory agencies like UNCST, National Institutes of Health, Office for Human Research Protections, study staff, study monitors, and their designees.

WHAT ARE MY RIGHTS?

Your participation is entirely voluntary and you are free to take part or withdraw at any time. This will not affect any medical care you may receive now or in the future. You may choose to answer some or all questions posed.

WILL I RECEIVE COMPENSATION?

You will receive UShs 50,000 for participating in the focus group or for the one-on-one interview. This is to help cover the cost of transportation and to compensate you for time spent on the study.

WHOM DO I CONTACT FOR QUESTIONS?

If you have questions about taking part in this research program, or about the information in this consent form, please contact the Principal Investigator Prof E. Katabira 0712-853410 or Dr. Mark Kaddumukasa 0772633475.

If you have questions about giving consent or your rights as a research participant, please contact the: Chairman School of Medicine Research and Ethics Committee. Prof Ponsiano Ocama; Tel: 0772421190 if these questions are not answered.

Summary of your rights as a participant in a research study

Your participation in this research study is voluntary. Refusing to participate will not alter your usual health care or involve any penalty or loss of benefits to which you are otherwise entitled. If you decide to join the study, you may withdraw at any time and for any reason without penalty or loss of benefits. If information generated from this study is published or presented, your identity will not be revealed. In the event new information becomes available that may affect the risks or benefits associated with this study or your willingness to participate in it, you will be notified so

that you can decide whether or not to continue participating. If you experience physical injury or illness as a result of participating in this research study, medical care is available at nearest government health center or elsewhere; however, the research study has no plans to provide free care or compensation for lost wages.

Disclosure of your study records

Efforts will be made to keep the personal information in your research record private and confidential, but absolute confidentiality cannot be guaranteed. The School of Medicine, Research and Ethics Committee, University Hospitals Case Medical Center Institutional Review Board may review your study records. If this study is regulated by the Food and Drug Administration (FDA), there is a possibility that the FDA might inspect your records. In addition, for treatment studies, the study sponsor and possibly foreign regulatory agencies may also review your records. If your records are reviewed your identity could become known.

Contact information

____ has described to you what is going to be done, the risks, hazards, and benefits involved. The Principal Investigator [Prof Elly Katabira] can also be contacted at [0712-853410]. If you have any questions, concerns or complaints about the study in the future, you may also contact them later.

If the researchers cannot be reached, or if you would like to talk to someone other than the researcher(s) about; concerns regarding the study; research participant's rights; research- related injury; or other human subject issues, please call the Chairman School of Medicine Research and Ethics Committee. Prof Ponsiano Ocama; Tel: 0772421190 if these questions are not answered.

Signature

Signing below indicates that you have been informed about the research study in which you voluntarily agree to participate; that you have asked any questions about the study that you may have; and that the information given to you has permitted you to make a fully informed and free decision about your participation in the study. By signing this consent form, you do not waive any legal rights, and the investigator(s) or sponsor(s) are not relieved of any liability they may have. A copy of this consent form will be provided to you.

x		
Signature of Participant		Date
x		
Printed Name of Participant		

x	
Signature of Participant	Date
x	
Printed Name of Participant	

Appendix C

SURVEY - REDUCING STROKE BURDEN IN UGANDA

Participant ID Initials

				-		

Section 1: Demographics

1.1 Interviewer name & code no.…………………………………

1.2 Date of interview: ☐☐ / ☐☐ / ☐☐☐☐ (dd / mm / yyyy)

1.3 Residence area:

1.4 Was written Informed Consent obtained? ☐ No ☐ Yes

If no, please do not proceed.

1.6 Gender: ☐ Male ☐ Female

1.7 Date of birth: ☐☐ / ☐☐ / ☐☐☐☐ (dd / mm / yyyy)

If year of birth not known ask or estimate age (years) |__|__|

1.8 Marital status:

☐ Married

☐ Single- never married

☐ Divorced

☐ Separated

☐ Widowed

1.9 Religion: ☐ Catholic

☐ Protestant

☐ Muslim

☐ Pentecostal

☐ Traditional

☐ Other………..

1.10 Highest level of education attained: ☐ None

☐ Primary (P1-7)

☐ Secondary (S1-6)

☐ Tertiary (University)

Section 2: Knowledge about stroke

2.1 What organ of the body is affected by stroke: ☐ Brain ☐ Heart ☐ Kidney ☐ Liver ☐ Lungs ☐ Don't know ☐ Other…………………

2.2 Is stroke preventable? :☐ Yes ☐ No

2.3 Can a person have stroke more than once? :☐ Yes ☐ No

2.4 Does stroke have an effect on daily activities like driving a car, dressing, use of the toilet and having a job? : ☐ Yes ☐ No

What do you believe causes a stroke? - (Tick all that apply)		
☐ Demons	☐ hypertension	☐ don't know
☐ Witch craft	☐ cigarette smoking	☐ Bad diet
☐ God's will	☐ Fatty foods	☐ alcohol
☐ Atherosclerosis	☐ high cholesterol	☐ Stress
☐ Angry ancestral spirits	☐ Obesity	
☐ Oral contraceptives	☐ lack of exercise	
☐ Inheritance		
☐ Others (please specify)…………………………………………………………		
**What do you believe are risk factors for stroke?**		

3.1 Do you know any risk factors for stroke? ☐ Yes ☐ No

If Yes, what are the risk factors for stroke that you know of? Please tick all that applies

☐ Old age ☐ hypertension

☐ Diabetes ☐ cigarette smoking

☐ Heart disease ☐ alcohol

☐ Atherosclerosis ☐ high cholesterol

☐ Obesity ☐ genetics (hereditary)

☐ Stress ☐ lack of exercise

☐ Poor hygiene ☐ headache or migraine

☐ Cancer ☐ oral contraceptives

☐ Bad diet ☐ tremors

☐ Others

Knowledge of stroke warning signs

3.2 Do you know any warning signs of stroke? ☐ Yes ☐ No

3.3 If Yes, what are the warning signs of stroke that you know of? Please tick all that applies

☐ Dizziness ☐ blurred or double vision or loss of vision

☐ Headache ☐ sudden difficulty in speaking or understanding or reading

☐ Tiredness ☐ fever/sweating

☐ Shortness of breath ☐ Chest pain or chest tightness

☐ Nausea/vomiting ☐ weakness of any part of the body

☐ Weakness of one side of the body ☐ paralysis of any part of the body

☐ Paralysis of one side of the body ☐fainting black out collapse

☐ Numbness tingling sensation or dead sensation of any body part

☐ Numbness tingling sensation or dead sensation of one side of the body

☐Others (please specify…………...

What would be your planned response to an event of stroke? (Tick all that apply)

☐ Call general practitioner or family doctor

☐ Ask family members or relatives to help

☐ Go to chemist for advice or medication

☐ Self-medication

☐ Ask friend or neighbors for help

☐ Go to hospital

☐ Visit community health center

☐ Visit alternative health care providers (herbal med, traditional healers),

☐ Seek spiritual healing (prayer)

☐ Combination of hospital and tradition

☐ Combination of hospital and faith

☐ Invite a Physiotherapist

☐ Others (please specify)

Sources of information about stroke (Tick all that apply)

What are your sources of information about stroke? Please tick all that applies

☐ Health care providers ☐ Friends and relatives

☐ Radio ☐ TV ☐ News papers

☐ Electronic media ☐ Others (please specify)…………

How would you like to receive information regarding stroke prevention?

From health workers (Doctors and nurses)	☐
Friends and relatives	☐
Newspapers	☐
Radio	☐
TV	☐
Church	☐
Community meetings	☐
Others (Specify)	☐

If a family member/relative has high blood pressure what advice would you give to this person?

(Tick all that apply)

Religiously take their anti-hypertensive medications	☐
Take them when they feel like	☐

Stop taking anti hypertensives ☐

Switch them to herbal therapies ☐

Recommend salt reduction ☐

Exercise ☐

Stop all work related activities ☐

Provide family support and care ☐

Recommend regular medical checks ☐

Interventions regarding stroke

What interventions would you suggest to a person with suspected stroke?

How would you rate immediate treatment of stroke?

Extremely important ☐

Somewhat important ☐

Important ☐

Can wait ☐

Not important ☐

Do you know about a therapeutic intervention window period where stroke signs and symptoms can be reversed?

Yes ☐

No ☐

Do you know any intervention therapies (Tick all correct responses)

Blood clot dissolving drugs ☐

Blood thinning drugs ☐

Blood pressure control ☐

Surgery ☐

Heart massage ☐

Natural herbs/therapies ☐

Traditional witch doctors therapy ☐

Other ☐

No treatment ☐

Would you participate in stroke prevention strategies?

Yes ☐

No ☐

Which intervention would you participate in? (Tick all that apply)

Health education classes ☐

Education and psychosocial counselling ☐

Active self-management ☐

Social support in management of hypertension ☐

Individual screening and risk management ☐

Community-wide interventions to address risk ☐

Does culture influence hypertension and stroke care?

Appendix D – Clinic evaluation

Reducing stroke burden in Uganda

Participant ID: Initials

				-		

1.1 Interviewer name & code no.…………………………………

1.2 Date of interview: __ __ / __ __ / __ __ __ __ (dd / mm / yyyy)

STROKE RISK FACTORS

Does the participant have past medical history for risk factors of a stroke?

		Yes No Unknown
1	Did a physician ever tell you that you have a stroke<br>If yes, go to 3	☐ ☐ ☐
2	Have you ever suffered from any of the following in a manner that was sudden?<br>Painless weakness on one side of your body,<br>numbness or a dead feeling on one side of your body, painless loss of vision in one or both eyes,<br>inability to understand what people are saying<br>inability to express yourself verbally or in writing?<br>Sudden severe headache with no known cause<br>If yes, how long did the symptoms last	☐ ☐ ☐<br><24hrs >24hrs<br>☐ ☐
3	Ever diagnosed or told that you have diabetes	☐ ☐
4	Ever diagnosed or told that you have hypertension	☐ ☐
5	Have you ever had a blood test for you lipid levels (Body fat)	☐ ☐ ☐

	If yes, were they high	☐ ☐ ☐
6	Have you ever had a heart attack	☐ ☐ ☐
7	Ever been diagnosed or told that you have heart disease	☐ ☐ ☐
8	Ever had heart surgery	☐ ☐ ☐
9	sickle cell disease (exclusion - will need to be asked at first visit)	☐ ☐ ☐
10	HIV/AIDS	☐ ☐ ☐

4c: Lifestyle/social activities

1 Tobacco use

Current use ☐ Yes ☐ No

If yes, what type of tobacco Cigarettes ☐ Other ☐

How many cigarettes per day 5-15 ☐ 16-20 ☐ >21 ☐

Former smoker ☐ Yes ☐ No

2 Alcohol use

Current alcohol consumption ☐ Yes ☐ No

CAGE screening questions

(1) Have you ever felt you ought to cut down on your drinking? ☐ Yes ☐ No

(2) Have people ever annoyed you by criticizing your drinking? ☐Yes ☐ No

(3) Have you ever felt guilty or bad about your drinking? ☐ Yes ☐ No

(4) Have you ever had a drink first thing in the morning to steady your nerves? ☐ Yes ☐ No

Interpretation; No suspected alcohol problem (if answer to all is no) ☐

Suspected alcohol problem (if one answer is yes) ☐

Alcohol abuse/dependency (if more than one answer is yes) ☐

3 Physical activity assessments

Are you engaged in any sport in the last 3 months? ☐ Yes ☐ No

If yes,

About how many times a week do you engage in the sport?

Rarely ☐ 1-2 times per week ☐ > 3 times per week ☐

Does this sport cause perspiration and breathlessness? ☐Yes ☐ No

Any current engagement in active physical exercise that caused perspiration and breathlessness? ☐Yes ☐No

If yes, specify activity …………………………………………………..

If no

About how many times a week do you engage in this activity?

Rarely ☐ 1-2 times per week ☐ > 3 times per week ☐

Means of transport? Walk ☐ Bicycle ☐ Taxi ☐ Drive☐

Motorcycle Ride☐ Ride on☐

4 Diet assessments

In a typical week, on how many days do you eat fruit, such as pineapple, mango, jackfruit, and passion fruit?

(Give number of **days**: 88 = don't know, 99 = refused to answer) |__|__| DIET1

If 0 days, go to

2. How many servings of fruit do you eat on one of those days? How defined?

(Give number of **servings**: 88 = don't know, 99 = refused to answer) |__|__| DIET2

3. In a typical week, on how many days do you eat vegetables such as cabbage, greens, carrots, and eggplant?

(Give number of **days**: 88= don't know, 99= refused to answer) ....|__|__| DIET3

If 0 days, go to question 5

4. How many servings of vegetables do you eat on one of those days?

(Give number of **servings**:88=don't know,99= refused to answer) ...|__|__| DIET4

5. In a typical week, on how many days do you eat starchy staples, such as posho, cassava, sweet potato and rice?

(Give number of **days**: 88 = don't know, 99 = refused to answer) |__|__| DIET5

If 0 days, go to question 7

6. How many servings of starchy staples do you eat on one of those days?

(Give number of **servings**:88 = don't know, 99 = refused to answer) |__|__| DIET6

7. In a typical week, on how many days do you eat matooke?

(Give number of **days**: 88 = don't know, 99 = refused to answer) |__|__| DIET10

If 0 days, go to question 9

8. How many servings of matooke do you eat on one of those days?

(Give number of **servings**: 88 = don't know,99= refused to answer) |__|__| DIET11

9. What type of oil or fat is most often used for food preparation in the household?

1 = Vegetable oil (e.g Bidco) 2 = Animal fat (Cowboy)

3 = Butter 4 = Margarine,

5 = Other 6 = None in particular

7 = None used 88=Don'tknow

|__| DIET12

If other type of fat or oil, specify________________________________ DIET12SP

10. When did you last eat anything? |__|__|: |__|__| DIET7

HR MIN (Give duration in **hours** and **minutes**)

11. When did you last drink anything, except water? HR MIN (Give duration in **hours** and **minutes**) |__|__|: |__|__| DIET8

12. Can you estimate how much salt is added to your food during cooking if you cook?

Unit: 1= Palm, 2=Teaspoon, 3=Pinch |__| DIET9U

Quantity: 00 = none 88 = don't know |__|__| DIET9

Family history assessment

Is the participant aware of presence of the following conditions among family members?

	Absent Present Unknown		
Stroke	☐	☐	☐
TIA	☐	☐	☐
Diabetes	☐	☐	☐
Hypertension	☐	☐	☐
Heart attack	☐	☐	☐
Other heart diseases	☐	☐	☐
Overweight/Obese	☐	☐	☐

Medication history

Record all the drugs the participant is currently using

Antihypertensives: ☐ captopril ☐ atenolol ☐nifedipine ☐aprinox ☐ Others…

Antiplatelet agents: ☐ aspirin ☐clopidogrel ☐ Others…………..

Anticoagulants: ☐ warfarin ☐heparin ☐ Others…………..

Antithrombotic: ☐streptokinase ☐ Others…………..

Lipid lowering drugs: ☐ atovastatin ☐ fenfibrate ☐ simvastatin ☐ Others…………..

Past and /or current oral contraceptives (women only):

☐Oral contraceptives ☐ Injectable contraceptives

Physical assessment

1 Height:cm ☐☐☐

2 Weight: ☐☐☐.☐ kg

5 Heart rate: ☐☐☐ bpm

6 Blood pressure (systolic/diastolic): ☐☐☐ / ☐☐☐ mm/Hg

Laboratory tests and results

Sample date: ☐☐ / ☐☐ / ☐☐☐☐ ☐ Not done

dd mm yyyy

CHEMISTRY			
	Value	Unit	
LDL-cholesterol		☐ mg/dL	
HDL-cholesterol		☐ mg/dL	
Triglycerides		☐ mg/dL	
Total Cholesterol		☐ mg/dL	
Blood sugar or HbA1c		☐ mg/dL	☐ mmol/l

HIV TESTING	
HIV test	☐ neg ☐ pos

New 8-item Morisky Medication Adherence Scale- MMAS-8

Do you sometimes forget to take your blood pressure medication?	☐ YES	☐ NO
In the last two weeks, was there any day when you did not take your high blood pressure medication?	☐ YES	☐ NO
Have you ever stopped taking your medications or decreased the dose without first warning your doctor because you felt worse when you took them?	☐ YES	☐ NO
When you travel or leave the house, do you sometimes forget to take your medications?	☐ YES	☐ NO
Did you take your high blood pressure medication yesterday?	☐ YES	☐ NO
When you feel your blood pressure is controlled, do you sometimes stop taking your medications?	☐ YES	☐ NO
Have you ever felt distressed for strictly following your high blood pressure treatment?	☐ YES	☐ NO
How often do you have difficulty to remember taking all your blood pressure medications?	Never/ Almost never / Sometimes/ Frequently/ Always	

SCREENING CONSENT FORM (ENGLISH)
Title of study: Reducing stroke burden in Uganda using Targeted Management Intervention (TEAM). A Randomized, Prospective 6 – month Controlled Trial.

You are being asked to take part in this study about identifying risk factors for stroke. The doctor in charge of this study at this site is Dr. Elly Katabira. Before you decide if you want to be a part of this study, we want you to know about the study.

This is a consent form. It gives you information about this study. The study staff will talk with you about this information. You are free to ask questions about this study at any time. If you agree to take part in this study, you will be asked to sign this consent form **first**. You will get a copy to keep.

This study is conducted by investigators from MakCHS, Nsambya, Mbarara University and Case Western Reserve University/UHCMC, Cleveland, OH (USA) and has been approved by both MakCHS and the UHCMC Institutional Review Boards.

WHY IS THIS STUDY BEING DONE?

This study is being done so that we can see what people in Uganda know about stroke and what the risk factors are.

WHAT THE STUDY IS ALL ABOUT?

Once you agree to participate, you will be asked to complete a questionnaire about what you know about stroke. The research staff will measure your blood pressure and they will ask you to go to a designated clinic within your area for an additional visit the next morning if your blood pressure is equal or above 140/90.

At the clinic visit, they will measure your blood pressure, and if your blood pressure is equal or higher to 140/90 at the two readings, the study staff will also take a sample of your blood through a vein in your arm (about 5ml or 1tsp). This blood will be used to test for the amount of fats and sugar in the blood, as well as for HIV. The study staff will also ask more questions about your general health, your medical history, any medications you may be taking and your lifestyle (diet, exercise, alcohol and tobacco use, etc.).

We expect that up to about 246 study participants will be enrolled with 123 in the study arm and 123 in the treatment as usual arm.

The study is interested in including the following below

4. Adults ≥ 18 years,
5. At risk for stroke defined by the following;
    a. High systolic BP >140 mmHg (assessed on at least 2 occasions at least 3 days apart and either criterion b or c as noted below:
    b. History of at least 1 other modifiable stroke risk factor including: diabetes, hyperlipidemia, obesity, smoking, alcohol dependent or sedentary lifestyle.
    c. History of stroke or transient ischemic attack within the past 5 years
6. Able to provide written informed consent to participate in the study.

However, those who have the following conditions will not be included into this study.

4. Individuals with sickle-cell disease (SCD)
5. Pregnant or lactating women,
6. Individuals with dementia using the Identification for Dementia in Elderly Africans (IDEA).

DURATION OF THE STUDY

If you are asked to participate in this study and you agree, you will be followed up for 6 months, with monthly visits. During this time, your blood pressure and blood samples for sugar, fats and kidney tests will be taken.

BENEFITS

There is no guarantee of benefits to any participants. However, subjects may benefit from knowing their risk of stroke and how to reduce the risk of various stroke risk factors using self-management.

If you agree to participate then a more detailed form will be given to you and you will be requested to sign that form.

I accept to continue with the study (TICK ONE)

YES ☐

NO ☐

Reason for objecting to participate in the study……………………………………………..

Screening consent forms – LUGANDA

Title of study: Reducing stroke burden in Uganda using Targeted Management Intervention (TEAM). A Randomized, Prospective 6 – month Controlled Trial.

OKWANJULA

Osabibwa okwetaba mu kunonyereza kuno okugenda okwekeneenya ebiviirako abantu okusanyalala. Omusawo akulira okunonyereza kuno ye Dr. Elly Katabira. Nga tonasalawo kwetaba mu kunonyereza kuno, twagala otegeere bye kukwatako.

Kino kye kiwandiiko ekiraga okukiriza kwo okwetaba mu kunonyereza. Kikubuulira ebyo byonna ebikwata ku kunonyereza era abakola ku kunonyereza kuno bajja kubikutegeeza. Oli wa ddembe okubuuza ebibuuzo kw'ebyo ebyekuusa ku kunonyereza obudde bwonna. Bw'onakiriza okwetaba mu kunonyereza kuno, ojja **kusooka** kusabibwa kusaako mukono era oweebweko kopi.

Kuno okunonyereza kukolebwa aba MakCHS ne Case Western Reserve University/UHCMC, Cleveland, OH (USA) nga kukiriziddwa MakCHS ne UHCMC Institutional Review Board.

LWAKI OKUNONYEREZA KUNO KUKOLEBWA?

Okunonyereza kuno kukolebwa okusobola okutegeera abantu mu Uganda kye bamanyi ku bulwadde bw'okusanyalala n'ebikuleeta.

KIKI KYE NINA OKUKOLA SINGA NEGATTA KU KUNONYEREZA KUNO?

Bwokiriza okwetaba mu kunonyereza, ojja kusabibwa okuddamu ebibuuzo ku by'omanyi ku bulwadde bw'okusanyalala. Abakola ku kunonyereza kuno bajja kupima puleesa yo era bakusabe okukyalira akalwaliro akakulinaanye amakya g'olunaku oluddako oddemu okupimibwa puleesa singa bakusanga nga puleesa yo yenkana oba okuyisa 140/90.

Ku ddwaliro, ojja kupimibwa puleesa naye singa osangibwa nga puleesa yo yenkana oba okusinga 140/90 ku kukeberebwa okw'emirundi ebiri, ojja kugibwaako omusaayi okuva mu musiwa gw'omukono (nga 5ml oba ekijiiko 1). Omusaayi guno gujja kukozesebwa okupima obungi bw'amasavu ne sukaali ebiri mu musaayi gwo n'okukebera akawuka akaleeta mukenenya. Abakola ku kunonyereza bajja kwongera okubuuza ebikwata ku bulamu bwo okutwaliza awamu, eby'obulwadde bwo, eddagala ly'okozesa n'engeri gye weyisaamu (eby'endya, dduyiro, omwenge n'okukozesa taaba, etc)

BANTU BAMEKA ABANETABA MU KUNONYEREZA KUNO?

Tusuubira abantu 246 okwetaba mu kunonyereza nga 123 mu kibinjja ekyo-kugesebwa ne 123 abokufuna obujjanjabi nga bulijjo.

Okunonyereza kugenda kwetaba mu bano wanmanga.

1. Abawezeza emyaka egisoba ku kumi namunana.
2. Abali kukabenja ku kufuna stroke okusinzila ku.
    a. Puleesa >140mmHg (epimidwa emirundu ebiri wakati we nnaku satu)
    b. Endwadde zino wamanga; ssukali, omugejjo, emassavu amngi mumusaayi, segeretti, omwenge
    c. Eya kubibwako puleesa mu myaka etaano egiwedde.
3. Na kirizza okwetaba mu musomo guno.

Bano abalina bino wamanga ssi bakwetaba mumusomo guno.

1. Abalwadde ba nalubiri
2. Abakyala b'embutto oba abayonsa
3. Abalwadde be wutta.

Ebbanga ly'Omusomo

Bwonoba nga okirizza okwetaba mumusomo guno, no kirizza, ogenda kumala emyezi mukaaga nga tukugoberera. Mukiseera kino, puleesa yo ne omusaayi gwo okupima sukaali, amassavu ne ensigo gujja kukeberwa.

WALIWO EBY'OKUGANYULWA MU KWETABA MU KUNONYEREZA?

Tewali kyakuganyulwa kyankomeledde eri ggwe ng'omuntu naye osobola okuganyulwa mu kumanya oba osobola okufuna obulwadde bw'okusanyalala. Osobola n'okuganyulwa mu kumanya obungi bwa sukaali n'amasavu byolina mu musaayi.

Ekigendelerwa ky'okukola okunonyereza kuno kya kumanya abantu kye bamanyi ku bulwadde bw'okusanyalala. Kino kisobolera ddala okuyamba abantu abalala mu maaso.

.

Bwoba nga okirriza okwetaba mumusomo guno, ojja kuwebwa ekiwandinko ekirara kwonosa omukono.

Nzikirizza okwetaba mu musomo ☐

Nedda ☐

Lwaki sisobodde kwetaba mumusomo…………………………………………………………
