# Supplementary material for: A 6-month, prospective randomized controlled trial of the TargetEd MAnageMent (TEAM) intervention vs. enhanced treatment as usual among Ugandans at risk for stroke
Source: PLoS One. 2025 Aug 22;20(8):e0330606. doi: 10.1371/journal.pone.0330606 (PMC12373184; doi:10.1371/journal.pone.0330606)
Supplement: Attachment — Submitted filename: Response to reviewers comments.docx [file pone.0330606.s005.docx]

7/9/2025

Dear Toshiki Maeda
Academic Editor
PLOS ONE

**Re: Response to editorial and reviewer’s comments for PONE-D-25-28874; A 6-month, prospective randomized controlled trial of the TargetEd MAnageMent (TEAM) Intervention vs. enhanced treatment as usual among Ugandans at risk for stroke.**

On behalf of the authors, I would like to thank you for the quick review and comments to improve our paper. We have made the suggested changes and corrections as shown below. We have resubmitted the manuscript in track changes and clean version for consideration.

We have included a rebuttal letter responding to each of the points raised by the academic editor and reviewers. .

Journal Requirements:

When submitting your revision, we need you to address these additional requirements.

1. Please ensure that your manuscript meets PLOS ONE's style requirements, including those for file naming. The PLOS ONE style templates can be found at https://journals.plos.org/plosone/s/file?id=wjVg/PLOSOne_formatting_sample_main_body.pdf and <https://journals.plos.org/plosone/s/file?id=ba62/PLOSOne_formatting_sample_title_authors_affiliations.pdf>

*We have revised the manuscript to meet the journal requirements including those for file naming.*

2. Please include a complete copy of PLOS’ questionnaire on inclusivity in global research in your revised manuscript. Our policy for research in this area aims to improve transparency in the reporting of research performed outside of researchers’ own country or community. The policy applies to researchers who have travelled to a different country to conduct research, research with Indigenous populations or their lands, and research on cultural artefacts. The questionnaire can also be requested at the journal’s discretion for any other submissions, even if these conditions are not met. Please find more information on the policy and a link to download a blank copy of the questionnaire here: https://journals.plos.org/plosone/s/best-practices-in-research-reporting. Please upload a completed version of your questionnaire as Supporting Information when you resubmit your manuscript.

*The PLOS questionnaire on inclusivity in global research has been completed and submitted.*

3. Thank you for stating the following financial disclosure:
“Martha Sajatovic and Elly Katabira received the award. “Research reported in this publication was supported by the National Institute Of Neurological Disorders And Stroke of the National Institutes of Health under Award Number R01NS118544. The content is solely the responsibility of the authors and does not necessarily represent the official views of the National Institutes of Health.”

Please state what role the funders took in the study. If the funders had no role, please state: "The funders had no role in study design, data collection and analysis, decision to publish, or preparation of the manuscript." If this statement is not correct you must amend it as needed.
Please include this amended Role of Funder statement in your cover letter; we will change the online submission form on your behalf.

The amended role of the funder has been included in the cover letter as indicated below.

*“The funders had no role in study design, data collection and analysis, decision to publish, or preparation of the manuscript.”*

4. Thank you for stating the following in the Competing Interests section:
“Dr. Sajatovic has research grants from Neurelis, Intra-Cellular, Merck, Otsuka, Teva and Alkermes, is a consultant to Alkermes, Otsuka, Janssen, Lundbeck, Teva and Neurelis and has received publication royalties from Springer Press, Johns Hopkins University Press, Oxford Press, and UpToDate.  The other authors have nothing to report.”

Please confirm that this does not alter your adherence to all PLOS ONE policies on sharing data and materials, by including the following statement: "This does not alter our adherence to PLOS ONE policies on sharing data and materials.” (as detailed online in our guide for authors http://journals.plos.org/plosone/s/competing-interests). If there are restrictions on sharing of data and/or materials, please state these. Please note that we cannot proceed with consideration of your article until this information has been declared.

Please include your updated Competing Interests statement in your cover letter; we will change the online submission form on your behalf.

*The competing interest statement has been corrected as indicated below.*

*“Dr. Sajatovic has research grants from Neurelis, Intra-Cellular, Merck, Otsuka, Teva and Alkermes, is a consultant to Alkermes, Otsuka, Janssen, Lundbeck, Teva and Neurelis and has received publication royalties from Springer Press, Johns Hopkins University Press, Oxford Press, and UpToDate.  The other authors have nothing to report. This does not alter our adherence to PLOS ONE policies on sharing data and materials.” (as detailed online in our guide for authors http://journals.plos.org/plosone/s/competing-interests)”*

5. We note that your Data Availability Statement is currently as follows: All relevant data are within the manuscript and its Supporting Information files.

Please confirm at this time whether or not your submission contains all raw data required to replicate the results of your study. Authors must share the “minimal data set” for their submission. PLOS defines the minimal data set to consist of the data required to replicate all study findings reported in the article, as well as related metadata and methods (https://journals.plos.org/plosone/s/data-availability#loc-minimal-data-set-definition).

For example, authors should submit the following data:

- The values behind the means, standard deviations and other measures reported;
- The values used to build graphs;
- The points extracted from images for analysis.

Authors do not need to submit their entire data set if only a portion of the data was used in the reported study.

If your submission does not contain these data, please either upload them as Supporting Information files or deposit them to a stable, public repository and provide us with the relevant URLs, DOIs, or accession numbers. For a list of recommended repositories, please see https://journals.plos.org/plosone/s/recommended-repositories.

If there are ethical or legal restrictions on sharing a de-identified data set, please explain them in detail (e.g., data contain potentially sensitive information, data are owned by a third-party organization, etc.) and who has imposed them (e.g., an ethics committee). Please also provide contact information for a data access committee, ethics committee, or other institutional body to which data requests may be sent. If data are owned by a third party, please indicate how others may request data access.

*We have included this statement in the manuscript. We will make the data and associated documentation available to users only under a data-sharing agreement (DUA) that provides for: (1) a commitment to using the data only for research purposes and not to identify any individual participant; (2) a commitment to securing the data using appropriate computer technology; and (3) a commitment to destroying or returning the data after analyses are completed. Beyond this, all the information generated from the various study data sets will be made available to the global community in open access journals indexed in pub med or via the internet. Qualified investigators can contact the study lead investigators:* [*Martha.sajatovic@uhhospitals.org*](mailto:Martha.sajatovic@uhhospitals.org) *or* [*kaddumark@yahoo.co.uk*](mailto:kaddumark@yahoo.co.uk) *for execution of an appropriate DUA. See page 24, lines 442 – 450.*

6. We note that you have included the phrase “data not shown” in your manuscript. Unfortunately, this does not meet our data sharing requirements. PLOS does not permit references to inaccessible data. We require that authors provide all relevant data within the paper, Supporting Information files, or in an acceptable, public repository. Please add a citation to support this phrase or upload the data that corresponds with these findings to a stable repository (such as Figshare or Dryad) and provide and URLs, DOIs, or accession numbers that may be used to access these data. Or, if the data are not a core part of the research being presented in your study, we ask that you remove the phrase that refers to these data.

*This statement has been deleted from the text, see page 19, line 330.*

7. Your ethics statement should only appear in the Methods section of your manuscript. If your ethics statement is written in any section besides the Methods, please move it to the Methods section and delete it from any other section. Please ensure that your ethics statement is included in your manuscript, as the ethics statement entered into the online submission form will not be published alongside your manuscript.

*The ethics section has been moved to the methods section, see page 8, lines 138 – 144.*

8. Please ensure that you refer to Figure 1 in your text as, if accepted, production will need this reference to link the reader to the figure.

*Figure 1 is included in the text, see page 12, line 234 – 235. .*

9. Please include captions for your Supporting Information files at the end of your manuscript, and update any in-text citations to match accordingly. Please see our Supporting Information guidelines for more information: <http://journals.plos.org/plosone/s/supporting-information>.

*The captions for the supporting files have been included at the end of the manuscript. See page 23, lines 417 – 419.*

10. Please review your reference list to ensure that it is complete and correct. If you have cited papers that have been retracted, please include the rationale for doing so in the manuscript text, or remove these references and replace them with relevant current references. Any changes to the reference list should be mentioned in the rebuttal letter that accompanies your revised manuscript. If you need to cite a retracted article, indicate the article’s retracted status in the References list and also include a citation and full reference for the retraction notice.

[Note: HTML markup is below. Please do not edit.]

*We have reviewed our reference list and it’s complete and correct.*

Reviewers' comments:

Reviewer's Responses to Questions

**Comments to the Author**

6+2
1. Is the manuscript technically sound, and do the data support the conclusions?

The manuscript must describe a technically sound piece of scientific research with data that supports the conclusions. Experiments must have been conducted rigorously, with appropriate controls, replication, and sample sizes. The conclusions must be drawn appropriately based on the data presented.

Reviewer #1: Yes

Reviewer #2: Yes

2. Has the statistical analysis been performed appropriately and rigorously?

Reviewer #1: Yes

Reviewer #2: Yes

3. Have the authors made all data underlying the findings in their manuscript fully available?

The [PLOS Data policy](http://track.editorialmanager.com/CL0/http:%2F%2Fwww.plosone.org%2Fstatic%2Fpolicies.action%23sharing/1/010f0197a6482134-ffd92bfc-cc65-4a19-87e7-3416ba236008-000000/9fGntRirk637VCvozPqQBzGaKytNhOKECL6IqMuKUl0=217) requires authors to make all data underlying the findings described in their manuscript fully available without restriction, with rare exception (please refer to the Data Availability Statement in the manuscript PDF file). The data should be provided as part of the manuscript or its supporting information, or deposited to a public repository. For example, in addition to summary statistics, the data points behind means, medians and variance measures should be available. If there are restrictions on publicly sharing data—e.g. participant privacy or use of data from a third party—those must be specified.

Reviewer #1: Yes

Reviewer #2: Yes

4. Is the manuscript presented in an intelligible fashion and written in standard English?

PLOS ONE does not copyedit accepted manuscripts, so the language in submitted articles must be clear, correct, and unambiguous. Any typographical or grammatical errors should be corrected at revision, so please note any specific errors here.

Reviewer #1: Yes

Reviewer #2: Yes

5. Review Comments to the Author

Please use the space provided to explain your answers to the questions above. You may also include additional comments for the author, including concerns about dual publication, research ethics, or publication ethics. (Please upload your review as an attachment if it exceeds 20,000 characters)

Reviewer #1: The manuscript is generally well written from a statistical perspective. The protocol was excellent as supplemental information providing the detail needed to explain the statistical design and analysis of this project. This included deriving a valid sample size and embellishment of the analysis detail in the manuscript. The sample size for the primary analysis is certainly adequate and accommodating of the secondary considerations of the trial. The ANOVA and MANOVA approach with repeated measures and added covariates certainly covers the routine aspects of the analysis expectations in this design. The authors did well in identifying the limitations of the analysis and the Team approach. There are several questions to be addressed:

1.What is 3wave? Is that simply the times, Baseline, 13 weeks and six months or something else?

*Response: 3 waves refers to the 3 time points of Baseline, 13 weeks, and six months. We have corrected this within the manuscript to time points within the manuscript, see page 12, lines 226 – 229.*

2. The investigators note that data that remain missing despite their retention efforts will be accommodated in their analyses and their impact evaluated through sensitivity analyses. Assuming MARS appears reasonable. However, why was there no consideration of the usual imputation strategies common in this case? Where is the sensitivity analysis, if any?

*We apologize for any misunderstanding.* *Given the relatively small amount of missing data, we did not conduct imputation analyses. However, we have compared baseline demographic and clinical characteristics of missing cases and did not find any substantive correlations with missing cases, suggesting no systematic bias in missing data.  These findings are consistent with data missing completely at random. We have included this under the data analysis section, see page 12, lines 231 – 235.*

3. Also, the authors note that they will also consider an AR (1) covariance model and compare model fits. There is really no discussion of covariate or confounder impact or model comparisons.

*We apologize for any misunderstanding. Regarding the AR (1) covariance model,  we chose to use RMANOVAs  as there were  a more parsimonious approach to analyzing the trend in the means of the primary outcomes, the major focus of the paper.*

Reviewer #2: The manuscript presents a well-conducted randomized controlled trial aimed at reducing stroke risk factors in high-risk individuals in a developing country setting. The study is technically sound, well written, and provides a pragmatic approach for mitigating stroke risk factors through an effective, community-based intervention.

Recommendation: Minor revisions

Specific Comments:

Line 95: It appears the authors intended to report a reduction in systolic blood pressure (SBP) and total serum cholesterol compared to baseline at 24 weeks, rather than the reverse. Please clarify this to avoid confusion.

*This has been corrected to reflect this, see lines 92 – 95.*

*“A 6-month, uncontrolled, prospective pilot study to establish feasibility, acceptability and preliminary efficacy of TEAM in Ugandans at high risk for stroke showed TEAM participants had significant reductions from baseline in mean systolic blood pressure (SBP) and in total serum cholesterol to 24-weeks levels.(9)”*

Intervention Description: The role and background of the PEDs (presumably stroke/TIA survivors acting as peer educators or supporters) should be more clearly described in the interventions section. This would enhance clarity and help readers better understand the implementation strategy.

*This section has been rewritten, see page 9, lines 153 -157.*

6. PLOS authors have the option to publish the peer review history of their article ([what does this mean?](https://track.editorialmanager.com/CL0/https:%2F%2Fjournals.plos.org%2Fplosone%2Fs%2Feditorial-and-peer-review-process%23loc-peer-review-history/1/010f0197a6482134-ffd92bfc-cc65-4a19-87e7-3416ba236008-000000/e3hRg0-tQg7Gi7RKJjgLcE6B0zsQolbP2Q-yLzQPth0=217)). If published, this will include your full peer review and any attached files.


If you choose “no”, your identity will remain anonymous but your review may still be made public.


**Do you want your identity to be public for this peer review?** For information about this choice, including consent withdrawal, please see our [Privacy Policy](https://track.editorialmanager.com/CL0/https:%2F%2Fwww.plos.org%2Fprivacy-policy/1/010f0197a6482134-ffd92bfc-cc65-4a19-87e7-3416ba236008-000000/thvm2RyDwgyHd4HGS1JIoLbHwtdE8h5hv6yNX35Q8Xs=217).

Reviewer #1: No

Reviewer #2: No

[NOTE: If reviewer comments were submitted as an attachment file, they will be attached to this email and accessible via the submission site. Please log into your account, locate the manuscript record, and check for the action link "View Attachments". If this link does not appear, there are no attachment files.]

While revising your submission, please upload your figure files to the Preflight Analysis and Conversion Engine (PACE) digital diagnostic tool, [https://pacev2.apexcovantage.com/](https://track.editorialmanager.com/CL0/https:%2F%2Fpacev2.apexcovantage.com%2F/1/010f0197a6482134-ffd92bfc-cc65-4a19-87e7-3416ba236008-000000/76PJHmx4fMTQ3UZSSxkV_xf1WRwZpbsl2zT3Moxaq6Y=217). PACE helps ensure that figures meet PLOS requirements. To use PACE, you must first register as a user. Registration is free. Then, login and navigate to the UPLOAD tab, where you will find detailed instructions on how to use the tool. If you encounter any issues or have any questions when using PACE, please email PLOS at [figures@plos.org](mailto:figures@plos.org). Please note that Supporting Information files do not need this step.
